# Identification of Cell Fate Determining Transcription Factors for Generating Brain Endothelial Cells

**DOI:** 10.1007/s12015-025-10842-7

**Published:** 2025-01-24

**Authors:** Roya Ramezankhani, Jonathan De Smedt, Burak Toprakhisar, Bernard K. van der Veer, Tine Tricot, Gert Vanmarcke, Bradley Balaton, Leo van Grunsven, Massoud Vosough, Yoke Chin Chai, Catherine Verfaillie

**Affiliations:** 1 Stem Cell Institute, Department of Development and Regeneration, KU Leuven, O&N IV Herestraat 49, Leuven, 3000 Belgium; 2 Department of Applied Cell Sciences, Faculty of Basic Sciences and Advanced Medical Technologies, Royan Institute, Academic Center for Education, Culture and Research, Tehran, Iran; 3 Liver Cell Biology Research Group, Vrije Universiteit Brussel, Laarbeeklaan 103, Brussels, 1090 Belgium; 4 KU Leuven Institute for Single Cell Omics (LISCO), KU Leuven-University of Leuven, Leuven, B-3000 Belgium; 5 Department of Regenerative Medicine, Cell Science Research Center, Royan Institute for Stem Cell Biology and Technology, Academic Center for Education, Culture and Research (ACECR), Tehran, Iran

**Keywords:** Blood brain barrier, Reprogramming Factors, BMECs, BBB Model, PSC, Differentiation

## Abstract

Reliable models of the blood-brain barrier (BBB), wherein brain microvascular endothelial cells (BMECs) play a key role in maintenance of barrier function, are essential tools for developing therapeutics and disease modeling. Recent studies explored generating BMEC-like cells from human pluripotent stem cells (hPSCs) by mimicking brain-microenvironment signals or genetic reprogramming. However, due to the lack of comprehensive transcriptional studies, the exact cellular identity of most of these cells remains poorly defined. In this study we aimed to identify the most likely master transcription factors (TFs) for inducing brain endothelial cell (EC) fate and assess the transcriptomic changes following their introduction into immature ECs. Therefore, we first generated PSC-derived immature ECs by transient overexpression of the TF, *ETV2*. Subsequently, by performing an extensive meta-analysis of transcriptome studies of brain and non-brain ECs, 12 candidate TFs were identified, which might fate immature ECs towards cells with brain EC features. Following combinatorial overexpression of these 12 TFs tagged with unique barcodes, single cell transcriptomics identified a subset of transduced cells that resembled mid-gestational human brain ECs. Assessment of the TF barcodes present in these cells revealed significant enrichment of the TFs *ZIC3*, *TFAP2C*, *TFAP2A*, and *DLX2*. These TFs might be useful to fate PSC-EC to BMEC-like cells, which could be incorporated in human in vitro BBB models.

**Graphical Abstract:**

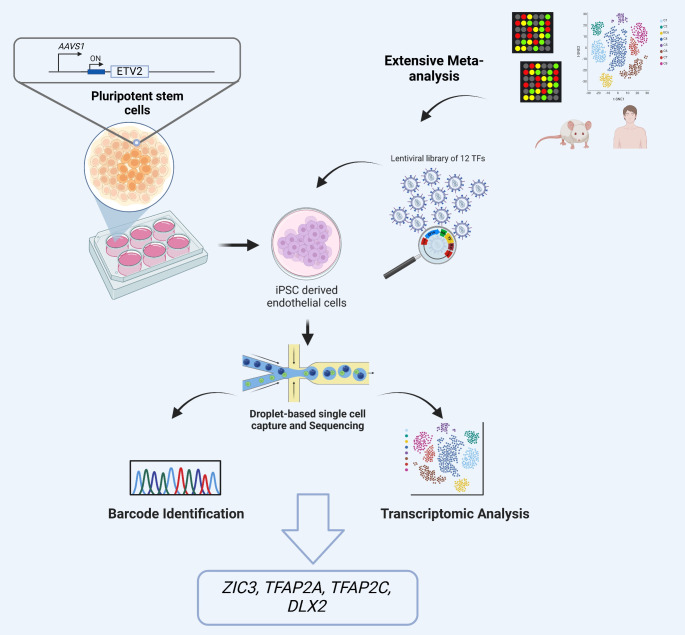

**Supplementary Information:**

The online version contains supplementary material available at 10.1007/s12015-025-10842-7.

## Introduction

To ensure a controlled and protected microenvironment in the central nervous system, (CNS), the blood brain barrier (BBB) is established during early fetal development. Through this process endothelial progenitor cells from preexisting blood vessels sprout into the developing neuroectoderm and generate new vessels, as the main anatomical units of the BBB [1, 2]. The newly formed vessels in the developing CNS acquire characteristics of typical brain microvascular endothelial cells (BMECs), such as expression of tight junctional (TJ) proteins and molecular transporters in a process called barriergenesis. The Wnt/β-catenin signaling pathway plays a major role in the process of TJ proteins and transporters such as SLC2A1 expression [2–4]. The full establishment of a functionalized BBB occurs by the third trimester of gestation [5]. This is the result of crosstalk of BMECs with pericytes, astrocytes, and neurons, which together form the complex structure of the neurovascular unit (NVU), which further enhances the unique barrier properties of BMECs [6]. Therefore, the BBB forms a strong constraint preventing toxin-induced brain damage while also impairing efficient drug delivery into the brain [7].

As a result, there is a great need for functional BBB models in the field of drug development, but also to model diseases that involve the BBB. The limitations inherent in animal-based models [8–10], as well as those based on immortal and primary cell lines, highlight the need for developing stable BBB models that maintain intact BMEC characteristics [11]. To that end, several 3D multicellular BBB models have been reported wherein human primary BMECs [12] or vascular organoid cultures [13, 14] have been combined with neural cells. In addition, significant efforts have also been made for the direct generation of BMEC-like cells from hPSCs. Since 2012, several differentiation strategies have been described to produce hPSC-derived BMEC-like cells by optimization of media that support the growth and functionality of putative BMECs in vitro [15–19]. Multiple studies incorporated these cells in co-cultures with pericytes and astrocytes [20] as well as in complex 3D model systems including organ-on-chip models [21]. However, a recent transcriptomic meta-analysis demonstrated that hPSC-derived BMEC-like cells as described in [15–19] lack the expression of multiple vascular marker genes including *CDH5* and *PECAM1*, along with several ETS transcription factors (TFs) (*ETS1*,* ETV6*, and *FLI*) [22]. Transduction of these cells with *ETV2*-, *FLI1-*, and *ERG*-encoding vectors resulted in the acquisition of endothelial identity but with attenuated barrier phenotype [22]. Alternatively, creation of PSC-derived brain ECs by activation of the Wnt pathway has been evaluated [23], as the importance of Wnt signaling is well documented in BBB establishment [3, 4]. In line with that, overexpressing TFs like *ZIC3* and *FOXF2*, which are downstream of the Wnt-β-catenin pathway, also promoted BMEC gene expression in human umbilical vein endothelial cells [24]. Alternatively, the combination of *ETS1*,* SOX7*,* SOX18* and either *TAL* or *LEF1* also induced a barrier resistance phenotype and expression of BMEC transporters in naïve hPSC-derived ECs (hPSC-ECs) [25], even if a full transcriptomic analysis was not done. To the best of our knowledge, no other studies have exploited TF-mediated induction of a brain EC fate from hPSCs.

In this study, to identify the possible master regulators for fating BMECs, we first generated hPSC-ECs by overexpression of *ETV2* [26–28] which creates cells expressing *bona fide* EC cell surface proteins such as PECAM1 and CDH5, as well as the transcripts for the ETS TFs, *i.e. ETS1*,* ETV6* and *FLI*. Because of the lack of knowledge about the intricate network of TF regulators that orchestrate brain EC differentiation and the absence of a comprehensive study that integrates multiple transcriptomics datasets that combines brain ECs with other EC subtypes, we next performed an extensive transcriptomic meta-analysis of BMECs versus other ECs, using multiple microarray datasets as well as single cell/nucleus (sc/sn)RNA-Sequencing (Seq) data. We identified 12 candidate master TFs for the induction of a BMEC fate. Combinatorial overexpression of the candidate TFs linked to unique barcodes [29] in immature ECs and scRNA-Seq revealed that a subset of transduced cells had a high similarity with developing human brain ECs in vivo. Furthermore, the directional trajectory of cells aligns with the transduction level, i.e. starting from lesser transduced to higher transduced states. Finally, by assessing the TFs barcodes present in cells with the most similarity to brain ECs, we identified *ZIC3* as well as *TFAP2C*,* TFAP2A*, and *DLX2* to be possible candidates for driving PSC-ECs to BMEC like cells.

## Materials and Methods

### Cell Culture

The human embryonic stem cell (hESC) line H9 (WA09) (with the ethical approval number of S67049), purchased from WiCell Research Institute (Madison, USA), was maintained on hESC qualified Matrigel^®^ (Corning) in E8 Flex medium (Thermo Fisher Scientific) and incubated at 37 °C, 5% CO_2_. Prior to passaging, 75% confluent hPSCs were detached with 0.5mM of EDTA (Thermo Fisher Scientific) in phosphate-buffered saline (PBS) (Thermo Fisher Scientific) and subsequently transferred to coated plates with hESC qualified Matrigel^®^ (Corning) diluted in DMEM/F-12 (Thermo Fisher Scientific) supplemented with RevitaCell (Thermo Fisher Scientific).

HEK 293T cells (CRL-3216) were purchased from ATCC and maintained in high glucose DMEM (Thermo Fisher Scientific) with 10% fetal bovine serum (FBS) (Thermo Fisher Scientific) and 1% penicillin/streptomycin (Thermo Fisher Scientific).

### Constructing hPSC line for Endothelial Differentiation

H9 PSCs were engineered via Flp/FRT system to express *ETV2* transcription factor upon doxycycline addition (iETV2-hPSCs), which is described elsewhere in detail [26].

### hPSC Differentiation towards Brain ECs

iETV2-hPSCs were first singularized with Accutase™ (Sigma–Aldrich) and subsequently seeded on growth factor-reduced Matrigel^®^ (Corning) coated 12-well plates in mTESR1 medium (StemCell Technologies) supplemented with RevitaCell (Thermo Fisher Scientific) at a density of ~ 35,000 cells/cm^2^. 24 h later (when the cells confluency was 40–60%), medium was switched to either liver differentiation medium (LDM) (composition in Supplementary Table 3A) and 5 µg/ml doxycycline (Sigma–Aldrich) for generating LDM-based ECs as described in [26], or neural maintenance medium (NMM) (Supplementary Table 3B) supplemented with 20 ng/ml bFGF (Biotechne (R&D)) and 5 µg/ml doxycycline for generating NMM-based ECs. After 48 h, in LDM group, medium was also supplemented with 2% FBS. For NMM-based ECs, media was further supplemented with 2% FBS on day 2, and 2% endothelial cell growth supplement (ECGS) (Sigma–Aldrich) on day 4, based on Chai et al. [30]. For splitting, cells were first dissociated with Accutase™ (Sigma–Aldrich) for 30 s in RT. Cells were harvested by gentle pipetting and plated on growth factor reduced Matrigel^®^ (Corning) coated 12-well plates in the respective media.

### Reverse-transcriptase Quantitative Polymerase Chain Reaction (RT-qPCR)

Total RNA was extracted using GenElute™ Mammalian RNA Extraction Kit (Sigma-Aldrich) and subsequently, cDNA was reverse transcribed with SuperScript^®^ III First-Strand Synthesis SuperMix (ThermoFisher Scientific). Platinum^®^ SYBR^®^ Green RT-qPCR SuperMix-UDG kit (ThermoFisher Scientific) was used for assessing gene expression through RT-qPCR. The reactions were set up as follows: 5 µl of mastermix, 1 µl of 2.5 µM forward and reverse primer mix, 2 µl of diluted cDNA (10 µg/µl), and 3 µl H2O. RT-qPCR was carried out under the following conditions: 95 °C for 10 min and 40 cycles of 95 °C for 15 s and 60 °C for 1 min. Gene expression values were normalized for GAPDH. Primer sequences are listed in Supplementary Table 4.

### Immunofluorescence Staining

Briefly, cells were fixed with 4% paraformaldehyde for 10–15 min, permeabilized with 0.2% Triton X-100 for ~ 10 min and subsequently blocked using 5% goat serum (Thermo Fisher Scientific) in PBS for 1 h in RT. Primary antibodies (CD31 (Agilent-M082329-2), VE-Cadherin (Abcam-ab33168), ZO1 (Proteintech- 21773-1-AP-S), and HA (Cell signaling Technology- CST 2367 S)) were then diluted according to the manufacturer’s protocol in Dako REAL Antibody Diluent (Dako) and added on the cells. Samples were incubated at 4 °C, overnight. The staining was proceeded by washing the cells with PBS and incubated with suitable secondary antibodies, for 1 h at 37 °C. Cells were then washed with PBS, and stained with Hoechst 33,342 (Thermo Fisher Scientific- H3570). Images were taken using Operetta™ CLS (PerkinElmer) imaging system or Nikon C2 laser scanning confocal microscope.

### Endothelial Tube Formation Assay

NMM/LDM-based ECs were dissociated using Accutase™ (Sigma–Aldrich) on day 9 of differentiation. 6–8 × 10^5^ cells were combined with 20 µl of ~ 10 mg/ml undiluted hESC qualified Matrigel^®^ (Corning) and inserted in 4-Chamber 35 mm glass bottom dishes (Cellvis) (20 µl per chamber). Matrigel drops were then solidified by incubation for at least 10 min at 37 °C. The related medium supplemented with 50 ng/ml VEGFA (Peprotech) was then added to the chambers. After 24 h, cells were fixed using 4% paraformaldehyde for ~ 20 min, permeabilized with 0.2% Triton X-100 in for ~ 30 min, blocked with 5% goat serum (Thermo Fisher Scientific) for 1 h and stained using anti-CD31 antibody (Agilent-M082329-2) at 4 °C, overnight. The samples were then carefully washed with PBS and stained with suitable secondary antibody for 1 h at 37 °C. After washing with PBS, nuclei staining was performed using Hoechst 33,342 (Thermo Fisher Scientific- H3570) and tubes were imaged with Nikon C2 laser scanning confocal microscope.

### Bulk RNA Sequencing Analysis

Total RNA was extracted from day 10 NMM-iETV2-EC (*n* = 3), upon stopping doxycycline addition on day 6 of differentiation. Single-end sequencing was performed on a NovaSeq SP 100 × 6 bp platform. Quality control of raw reads was performed with FastQC v0.11.7 [31]. Adapters were filtered with ea-utils fastq-mcf v1.05 [32]. Splice-aware alignment was performed with HISAT2 [33] against the reference genome hg38 using the default parameters. Reads mapping to multiple loci in the reference genome were discarded. Resulting BAM alignment files were handled with Samtools v1.5 [34]. Quantification of reads per gene was performed with HT-seq Count v0.10.0, Python v2.7.14 [35].

### Transcriptomic Meta-analysis

To identify candidate transcription factors (TFs) with the highest potential for driving the fate of immature PSC-ECs towards brain ECs, several bioinformatics pipelines were employed to analyze single cell/nucleus (sc/n)RNA-Sequencing (Seq) as well as micro-array expression data from multiple publications and datasets. Microarrays data were retrieved from ArrayExpress database at EMBL-EBI (www.ebi.ac.uk/arrayexpress) of brain samples (including ECs). All microarray analyses were done using *cenTFinder* R package, in January/February 2021. The detailed process has been described elsewhere [26]. Briefly, key words, i.e. “endothelial cell AND Mus musculus” “blood brain barrier AND Mus musculus”, “BBB AND Mus musculus”, and “brain endothel AND Mus musculus” for brain including ECs, as well as “endothelial cell AND Homo sapiens” for non-brain ECs, were used for querying the ArrayExpress database to retrieve relevant accession numbers. Next, accession numbers were retained when they corresponded to Affymetrix arrays labelled with biotin. New URLs were constructed to download CEL files of the selected arrays. The files were further filtered to include relevant samples, using the keywords “endothelial”, “BBB”, “brain”, and “blood brain barrier endothelial cell” for brain including ECs and “endothelial” for non-brain ECs. To exclude irrelevant samples, the keywords “oma”, “tumor”, “tumour”, “cancer”, and “malignant” were used, according to De Smedt et al. [26]. Next, platform type information was extracted from all CEL files in order to identify the respective array annotations. The downstream analysis was then performed with 10,205 genes and 193 samples for brain including ECs and with 16,248 genes and 279 EC arrays for non-brain ECs [26]. The total number of genes in non-brain EC dataset was restricted to 9,000 to save on computational time (without removing any transcription factor), based on De Smedt et al. [26]. The normalization and batch correction were done using *CenTFinder* R package.

Next, for analyzing the co-expression networks, Weighted Gene Correlation Network Analysis (WGCNA) was performed on the expression matrices and signed networks were constructed [36]. Soft thresholding powers from 1 to 30 were considered. Cluster dendrograms were created based on the topological overlap matrix with a minimum cluster size of 30. Co-expression modules were merged with a dissimilarity less than 0.3. Further analyses, i.e. Gene Ontology (GO) (i.e. Biological Process, Cellular Component, and Molecular Function) were performed on each module gene sets. Subsequently, to identify the relevant module in each endothelial cell subtypes including brain ECs, we used Gene Set Variation Analysis (GSVA) and scored the module gene sets for each sample. The cisTarget motif database, ‘*h19-tss-centered-10 kb-7species.mc9nr*’, was downloaded in February 2021 from https://resources.aertslab.org/cistarget/ to perform RcisTarget analysis and to identify the significantly enriched TF binding motifs within modules [37]. TFs with a Normalized Enrichment Score (NES) higher than 3 were considered. All the markers were identified using the *plotMarkers* function in *cenTFinder* package with the default settings.

For sc/nRNA-Seq analysis, the expression count matrices were obtained from the relevant provided links (Supplementary Table 5). Next, the expression matrices were filtered based on a unique cut off for the number of gene counts across all cells in each specific dataset. As each dataset possessed a unique amount of gene coverage, we determined specific cutoffs for individual studies. Genes with at least 10 counts [38], 50 counts [39, 40], 100 counts [41–43], 300 counts [44], were retained for the related dataset. We then further removed genes if they were expressed in less than 1% of all cells or absent in the gene motif ranking database (used for Single-Cell Regulatory Network Inference and Clustering (SCENIC)) analysis). *Seurat* R package was used for clustering. We used a custom-made script for identification of marker genes using the Wilcoxon rank sum test (min.pct = 0.25, fc.threshold = 1.2).

SCENIC analysis [37] was used to identify the regulons from each dataset. Next, we scored identified regulon’s activity in each cell or nucleus (from sc/nRNA-Seq studies) and in each microarray sample using the expression matrices of the four individual datasets, as follows. Given the high gene coverage in Allen human [40] and mouse [42] Brain Map datasets and the study of Fan et al. [43], the expression matrices of common genes in EC clusters of these studies were used to build gene expression rankings for calculating the regulon’s activities. Similarly, the expression matrices from microarray data (including brain containing-ECs and non-brain ECs), as well as Kalucka et al. study [41] were used to build individual gene expression rankings to calculate each regulon’s activity. The ranking for the non-brain ECs was reversed as we were interested in regulons that were underrepresented in this dataset.

Additionally, the median activity in brain ECs from sc/snRNA-Seq studies [38–40, 42–44] and human non-brain ECs from the microarray datasets was calculated from the AUC values using the *quantile* function. The average activity in murine brain ECs from microarray datasets were calculated using the *mean* function on AUC values in only brain ECs. To calculate how informative is the regulons, the mutual information (MI) between the computed AUC values and vascular bed position was determined using the *mutinformation* function [41].

For all the studies included mouse gene symbols, gene lift-over analysis was performed using the biomaRt package [45] in R and the Ensembl database to convert the gene symbols to their human orthologues.

### Plasmid Construction and Library Preparation

EF1a_mCherry_P2A_Hygro plasmid (Supplementary Table 6) was used to construct the lentiviral backbone. Briefly, the mCherry_P2A_Hygro fragment was first removed and an HA tag (synthesized by IDT DNA technologies) was inserted via ligation in a reaction mix included 4 ng of fragment, 50 ng of the digested vector, 1 µl of T4 DNA ligase, and 2 µl of ligase buffer in a total volume of 20 µl (New England Biolabs) and incubated at 22 °C for 1 h. The HA tag was followed by XbaI and HpaI restriction sites to allow cloning of each TF, immediately downstream of the tag. The final plasmid was digested with *XbaI* and *HpaI* restriction enzymes (Thermo Fisher Scientific), separated and extracted from a gel, and used to assemble TF and barcode.

The coding regions of the different TFs were either amplified from the related plasmids, a human cDNA pool, or synthesized as gBlock (IDT DNA technologies) (*PRDM5* and *PAX5*) using Q5 Hot Start High-Fidelity DNA polymerase (New England Biolabs). The vectors used for amplifying the CDSs of *DLX2*, *TFAP2C*, *SPIB*, *FOXF2*, *FOXQ1*, *TCF7*, *TFAP2A*, *KLF4*, *ZIC3*, and *HNF4A* are listed in Supplementary Table 6. Each TF-harboring fragment was integrated into the be backbone using Gibson assembly along with a unique 20 bp barcode containing DNA. The unique 20 bp barcode was located 196 bp upstream of the 3’-LTR region in the final assembled constructs, allowing barcode capture during sample processing.

To construct the eGFP and BFP-containing lentiviral vectors, the mCherry sequence was first removed from EF1a_mCherry_P2A_Hygro plasmid (Supplementary Table 6) and the CDSs of eGFP and BFP (which were amplified from pUC19_SalI_eGFP_BamHI and pLVX-EF1a-BFP-IRES-HYG plasmids, which were available in lab respectively) were inserted by Gibson assembly. For constructing the vector with no fluorochrome, only mCherry sequence was removed and the plasmid was re-circularized using a 60 bp oligonucleotide, including flanking sequences of the digested site, by Gibson assembly.

All PCR reactions were set up as follows: 10 µl of Q5 buffer, 1 µl of 10 µM dNTP, 5 µl from each primer (5 µM), 50 ng of template DNA, 0.5 µl of Q5 DNA polymerase, 10 µl of Q5 enhancer, and H_2_O up to 50 µl. PCR was carried out under the following conditions: 98 °C for 5 min, 35 cycles at 98 °C for 30 s, at temperature annealing for 30 s, and 72 °C for 1 kb per min, and a final 10 min extension at 72 °C. All the digestion processes were performed at 37 °C for 2 h. All PCR purifications were performed using the PureLink PCR Purification Kit (Thermo Fisher Scientific) and all the gel purification were performed using the PureLink™ Quick Gel Extraction Kit (Thermo Fisher Scientific), where applicable. All the Gibson assembly reactions were performed using 2X Gibson assembly master mix (New England Biolabs), and H2O up to 20 µl, followed by an incubation time of 1 h at 50 °C. All the products from ligation and Gibson assembly reactions were subsequently transformed into competent E.coli. After picking the growth clones and culturing them for 12 h, all the plasmids were then purified using a PureLink™ HiPure Plasmid Miniprep Kit (Thermo Fisher Scientific) and Sanger sequenced to verify the correct assembly and potential mutations. All assembled plasmids were further amplified in large-volume bacterial cultures and purified with a PureLink HiPure Plasmid Filter Maxi Prep Kit (Thermo Fisher Scientific).

### Transfection

To assess the expression of the barcoded TFs at the protein level, HEK-293T cells were dissociated with Accutase™ (Sigma–Aldrich) and seeded at a density of 1 × 10^4^ cells per well of black, flat bottom 96 well plates (Greiner Bio-One). 24 h later, transfection was done by adding 200 ng of plasmid DNA to 9 µl of HEK medium and mixed well. Subsequently, 0.6 µl of FuGENE^®^ HD Transfection Reagent (Roche Applied Science) was added to the mixture, pipetted well and incubated in RT for 15 min. The final mixture then was added to the cells and the medium was changed after 12 h.

### Viral Vector Production

HEK-293T cells with low passage numbers, typically between 7 and 9, were used to produce and package the lentiviral vector particles. A mixture of 18 µg of psPAX2, 6 µg of pMD2.G (Supplementary Table 6), and 18 µg of individual lentiviral vectors added to 500 µl of pre-warmed Opti-MEM (Thermo Fisher Scientific). In addition, 105 µl of FuGENE^®^ HD Transfection Reagent (Roche Applied Science) was added to the mixture, mixed well by pipetting, and incubated for 15 min at room temperature. The final mixture was then added to 20 ml of pre-warmed medium and added to HEK cells, that were 70–80% confluent. 48 h later, the supernatant was collected, centrifuged 5 min at 300 g to remove cell debris, and was filtered using 0.45 μm Acrodisc^®^ syringe filters (PALL). The supernatant was further concentrated using Vivaspin^®^ 20 ml centrifugal concentrators (Fisher Scientific) at 3500 rpm for 1 h at 4 °C. MOI calculation was performed using Lenti-X™ p24 Rapid Titer Kit (Takara).

### Viral Vector Transduction

On day 4 of differentiation, NMM-iETV2 ECs were seeded at a density of ~ 65,700 cells/cm^2^ in a 12-well plate. On day 6, NMM-iETV2 ECs were transduced with TF-harboring lentiviral particles at the highest transduction titer where no toxicity was observed in the control wells (based on [29]) in combination with 5 µg/ml Protamine sulfate (Fisher Scientific) to increase the transduction efficiency. For all the transductions, medium was changed 24 h after the transduction.

### Single Cell Library Preparation

Four days after transduction, NMM-iETV2 ECs were dissociated using Accutase™ (Sigma–Aldrich) and suspended in PBS + 0.04% bovine serum albumin (BSA). Cell viability was assessed using the Luna FL™ Cell Counter (Logos Biosystems, South Korea) according to the manufacturer’s instructions. The single-cell suspension was resuspended at an estimated final concentration of 1000 cells/µl and loaded on a Chromium GemCode Single Cell Instrument (10x Genomics) to generate single-cell gel beads-in-emulsion (GEM). The scRNA-Seq libraries were prepared using the GemCode Single Cell 3’ Gel Bead and Library kit, version NextGEM 3.1 (10x Genomics) according to the manufacturer’s instructions. The cDNA content of pre-fragmentation and post-sample index PCR samples was analyzed using the Fragment Analyzer (Agilent). All sequencing libraries were loaded on an Illumina NovaSeq6000 flow cell at VIB Nucleomics core with sequencing settings according to the recommendations of 10x Genomics.

### Barcode Amplification

Barcodes were amplified from cDNA generated by the Single Cell 3’ Reagent Kit v3.1 (10X Genomics) using a protocol modified from Parekh et al., 2018. Amplification was performed in three separate 50 µl reactions using 2 µl of the cDNA as input per reaction with Kapa Hifi Hotstart ReadyMix (Roche). The PCR primers were Read1_FW: CTACACGACGCTCTTCCGATCT and Read2_TF_REV: GTGACTGGAGTTCAGACGTGTGCTCTTCCGATCTAGAACTATTTCCTGGCTGTTACGCG. The parameters for the thermocycler were 95 °C for 3 min, followed by 26 cycles of 98 °C for 20 s, 65 °C for 15 s and 72 °C for 30 s. The final extension was 72 °C for 5 min. The three reactions were pooled, and amplicons were purified using Agencourt AMPure XP beads at 0.8 ratio. The second index PCR was performed on 100 ng of purified amplicon, with Kapa Hifi Hotstart ReadyMix (Roche), using a Dual index TT Set A primer set (10X Genomics). The thermocycler parameters were 95 °C for 3 min, followed by 6 cycles of 98 °C for 20 s, 54 °C for 30 s and 72 °C for 30 s. Final extension was 72 °C for 5 min. The PCR product containing the final barcode library was purified using Agencourt AMPure XP beads at 0.8 ratio and quantified by Qubit dsDNA HS assay (Thermo Fisher Scientific) and loaded on Illumina NovaSeq6000.

### Quality Control, scRNA-Seq Data pre-Processing

The Cell Ranger pipeline (10x Genomics, version 6.1.2) was used to perform sample demultiplexing and to generate FASTQ files for read 1, read 2 and the i7 sample index for the gene expression library. Read 2 of the gene expression libraries was mapped to the reference genome (GRCh38.99) using STAR. Subsequent barcode processing, unique molecular identifiers filtering, and gene counting was performed using the Cell Ranger suite version 6.1.2.

#### Genotype Deconvolution

To assign the correct TF barcode to each cell, the code from Parekh et al., 2018 was used (https://github.com/yanwu2014/genotyping-matrices) and modified for the barcode structure of v3.1 chemistry.

### Normalization, Clustering, and Differential Gene Expression of scRNA-Seq Data

To further filter the low-quality cells, normalize, scaling and clustering, we used the *Scater* package and the Seurat pipeline. The gene count matrix was filtered by removing cells with less than 200 expressed genes and the genes that are expressed in less than 3 cells. An additional quality control was done using *Scater* package in a univariate and multivariate ways by filtering the cells with very low or high total number of counts/transcripts, (library size (number of median absolute deviations was defined as 5)), the cells with low or high number of expressed genes (number of median absolute deviations was defined as 5), and the cells with a very high percentage of mitochondrial transcripts (number of median absolute deviations was defined as 5).

A Seurat object was created from the counts and metadata and the count matrix was log normalized. The most highly variable genes were determined using the FindVariableFeatures function with the default parameters. To select the largest source of variability, the data was scaled, and 50 principal components (PCs) were calculated based on the scaled normalized counts of overdispersed genes through PCA analysis (using RunPCA function with the default parameters). Based on the heatmap for visualization of top genes contributing to each PC, the first 15 PCs were selected to identify the nearest neighbors (using the FindNeighbors function with the default parameters) and then to produce an S Nearest Neighbors (SNN) graph with the resolution of 0.8 (using FindClusters function with the default parameters) to find the cell clusters. Subsequently, results were visualized using Uniform Manifold Approximation and Projection (UMAP) on the first 15 PCs using the RunUMAP function. Additionally, the information regarding the presence of each TF barcode was added to the metadata of the final Seurat object. Finally, DEGs in each cluster were determined using FindAllMarkers function (min.pct = 0.01, min.cells.feature = 3, min.cells.group = 3, and test.use = “wilcox”). Subsequently only the DEGs with an average LogFC > 0.25 and P-Value < 0.05 were selected for the downstream analysis. To identify the barcodes that were significantly enriched in each cluster, *permutation_test* function (with the default settings) from *scProportionTest* package was used. Subsequently, *permutation_plot* function (log2FD_threshold = log2(1.275)) was used to depict the point-range plots [46].

### Enrichment Analysis

To identify clusters with the most similar transcriptome to brain (microvascular) ECs, the list of upregulated genes in each cluster was used for GO enrichment analysis (using Biological process, Molecular Function, and Cellular component 2021 gene set libraries (p-values < 0.05)), pathway enrichment analysis (using Panther 2016 database (p-values < 0.05)) and cell type enrichment analysis (using Allen Brain Atlas 10X SCRNA 2021 database (p-values < 0.05)) through Enrichr database (https://maayanlab.cloud/Enrichr/).

### Integration Analysis and Trajectory Inference Analysis

To identify the cell clusters most similar to in vivo human brain (microvascular) ECs, the data was merged with several developing human brain as well as adult single cell RNA seq datasets [43, 47, 48], in vitro cultured primary human BMECs [22], day 11 induced BMECs (based on Lippmann et al.) [16, 22], rECs (reprogrammed Epi-iBMECs with *ETV2*, *ERG*, and *FLI1*) [22], and fetal liver [49] through the merge function and the default settings. The merged data was then normalized and upon finding the highly variable genes, scaling, and performing the PCA analysis, the clusters were identified. Subsequently, with the aim of identification of anchors between clusters and performing batch correction, the FindIntegrationAnchors function was used, employing first 30 dimensions. Next, these anchors were used to integrate the two datasets with IntegrateData function with default settings. The integrated data was normalized, and upon finding the highly variable genes, scaling, and performing the PCA analysis, clusters were identified. The resulted object was then used for downstream analysis.

Additionally, the merged data of fetal brain ECs from different developmental stages [43, 47], adult brain ECs [48], and the cluster of transduced PSC-derived cells with BMEC-like cells was used to infer the linear developmental chronologies using the SCORPIUS package [50]. The *reduce_dimensionality* function (dist = spearman, ndim = 3) was used to perform the dimensionality reduction employing the integrated expression matrix of in vivo brain ECs and the expression matrix of cluster 1^T^ as well as un-traduced cells separately. To infer linear trajectory through space, from fetal to adult brain ECs we utilized *infer_trajectory* function and positioned the (un)-transduced ECs on the trajectory.

### RNA Velocity Analysis

To infer transcriptional dynamics and predict cell fate decision via pseudotime trajectories in our scRNA-Seq data, RNA Velocity analysis was performed using the Python package scVelo [51] and under the instructions of Bergen et al. (2020) (https://scvelo.readthedocs.io/). Briefly, the filtered and normalized Seurat object was converted to anndata format and was used the input of analysis. Subsequently, the *velocyto* command line tool was used to calculate the spliced and unspliced counts matrix from the CellRanger output directory. Subsequently, RNA velocity was computed.

### SCENIC Analysis

To determine the TF gene regulatory network (GRN)/regulon’s activity in each cell and to find cell cluster master TFs in cell clusters, SCENIC pipeline [37] was used. In summary, the filtered and normalized gene expression matrix was filtered to remove genes that are not present in the *hg19-tss-centered-10 kb-7species.mc9nr.feather* database. The final filtered read counts was then used as the input for GRNBoost2 as a part of arboreto (v 0.1.6) in python, to identify the co-expression pattern between the TFs and their potential targets. Targets with only positive correlations were then selected from the inferred network and the final network was then assessed to calculate motif enrichment for each TF-module and therefore to identify the possible regulons, using the *RcisTarge*t package (v 1.15.3) [37], based on a motif dataset (hg19-tss-centered-10 kb-7species.mc9nr.feather). Subsequently, the regulon activity in each cell was calculated using AUCell [37] with the default threshold. Finally, upon clustering cells based on the original umap coordinates from the initial analysis, the regulon’s activity was plotted for each cluster.

### Statistical Analysis of RT-qPCR

All experiments were performed in biological triplicate and the data are shown as mean ± s.d. To assess the statistical significance, two-way ANOVA with Sidak’s post hoc test was used (GraphPad prism v.10). p-values < 0.0332 (*), *p* < 0.0021 (**) and *p* < 0.0002 (***) and *p* < 0.0001 (****) were considered statistically significant.

## Results

### Neural Maintenance Medium (NMM) Combined with ETV2 Overexpression can Support the Differentiation of ECs from hPSCs

**Fig. 1:**
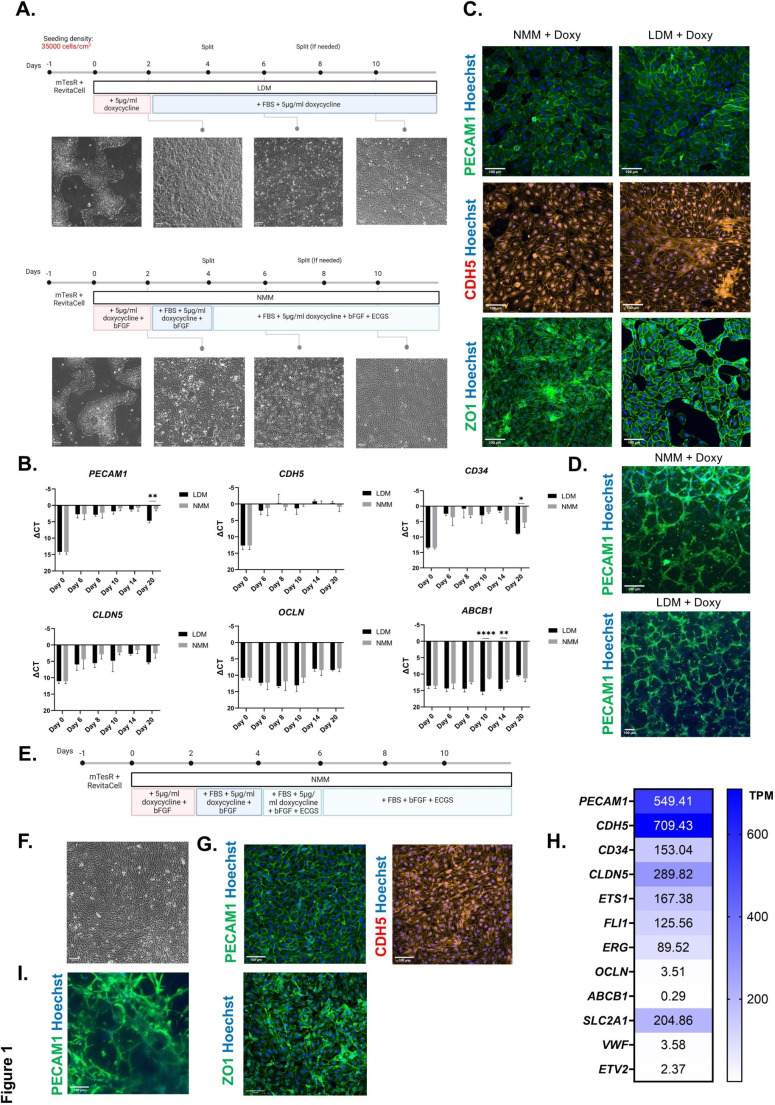
Generation and characterization of human pluripotent stem cell (hPSC)-derived ECs.**(A)** Schematic diagrams of protocols to differentiate hPSCs into ECs using LDM (Top) and NMM (Bottom). Phase contrast images show morphological changes of cells on days 0, 2, 6, and 10 of differentiation. **(B)** Gene expression analysis of endothelial lineage marker genes (*i.e. PECAM1*, *CDH5*, and *CD34*) and brain EC marker genes (*i.e. CLDN5*,* OCLN*, AND *ABCB1*) until day 20 of differentiation (*N* = 3 biological replicates,; mean *±* s.d., two-way ANOVA: p-values < 0.0332 (*), *p* < 0.0021 (**) and *p* < 0.0002 (***) and *p* < 0.0001 (****)). **(C)** Confocal microscopic images of day 10 LDM- and NMM-ECs for PECAM1 (green), ZO1 (green), CDH5 (red), and Hoechst (blue). **(D)** Tube formation of day 10 LDM- and NMM-ECs in Matrigel, stained for PECAM1 (green) and Hoechst (blue). **(E)** Schematic diagram of protocol used to differentiate hPSCs into ECs after transient overexpression of *ETV2* by doxycycline, until day 6, and 4 additional days in culture without doxycycline. **(F)** Phase contrast images of NMM-EC on day 10 with removal of doxycycline from day 6 of differentiation. **(G)** Confocal microscopic images of NMM-EC on day 10 with removal of doxycycline from day 6 of differentiation, for PECAM1, ZO1, CDH5 (red), and Hoechst (blue). **(H)** Heatmap of Transcripts Per Kilobase Million (TPM) values for (brain) endothelial marker expression of NMM-EC on day 10 with removal of doxycycline from day 6 of differentiation (*N* = 3 biological replicates). **(I)** Tube formation of NMM-EC on day 10 with removal of doxycycline from day 6 of differentiation in Matrigel stained for PECAM1(green) and Hoechst (blue). bFGF: Basic fibroblast growth factor, EC: Endothelial cell, ECGS: Endothelial cell growth supplement, FBS: Fetal bovine serum, PSC: Pluripotent stem cells, LDM: Liver differentiation medium, NMM: Neural maintenance medium, (Scale bars, 100 μm)

We previously described creation of endothelial cells from PSCs by induced overexpression of *ETV2*, and when combined with induction of PU.1 such ECs could be fated to cells with some liver sinusoidal endothelial cell features [26]. Endothelial differentiation was performed in so-called Liver Differentiation Medium (LDM) as this was also suitable for culturing iPSC-derived hepatocyte-like cells. As creation of a blood brain barrier model will require that brain microvascular endothelial cells (BMECs) are cocultured with neural cells, we tested if differentiation of hPSCs towards ECs is also possible using Neural Maintenance Medium (NMM), which is used commonly to generate neurons and glial cells. Therefore, we differentiated hPSCs by overexpression of *ETV2* in either LDM or NMM and evaluated EC generation (Fig. 1A). RT-qPCR was used to assess the endothelial lineage marker genes (*PECAM1*, *CD34*, *CDH5*). No major differences were seen between ECs generated in NMM medium compared with LDM (Fig. 1B). Immunofluorescence (IF) staining confirmed that similar numbers of cells stained positive for PECAM1 and CDH5 as well as the junction protein ZO1 (Fig. 1C and Supplementary Fig. 1A). Although *CLDN5* transcript levels were high in both conditions (Fig. 1B), transcripts for two other brain EC marker genes, *i.e. OCLN* and *ABCB1*, remained low in both protocols (Fig. 1B), and positive staining cells were not detected for these markers (Data not shown). To further demonstrate EC identity, we performed a Matrigel^®^ (Corning) tube assay, and ECs from both culture conditions were able to form tubes (Fig. 1D). Therefore, we selected NMM for the culture of putative BMECs.

When we evaluated *ETV2* expression during the early developmental stages of fetal brain (~ 6–23 gestational weeks) we found that this TF was expressed at low levels (0.003–0.059 TP10K) in brain ECs (Supplementary Fig. 1B-F), which is consistent with the temporarily high expression of this master TF during very early embryogenesis [52]. Therefore, we tested if removing doxycycline on day 6 from the EC cultures, and hence reducing *ETV2* expression, would affect the EC differentiation (Fig. 1E). Removal of doxycycline on day 6 did not affect the morphological characteristics of the cells on day 10 (Fig. 1F). In addition, IF staining confirmed the persistent homogenous presence of PECAM1, CDH5, and ZO1 positive cells (Fig. 1G), and crucial TFs for the EC lineage, *i.e. ETS1*,* FLI1* and *ERG* remained expressed as shown by bulk RNA sequencing (Fig. 1H). Finally, the cells retained tube formation capacity (Fig. 1I).

### Transcriptomic Meta-analysis Identified 12 TFs as Potential Master Regulators for Driving BMECs Fate

**Fig. 2:**
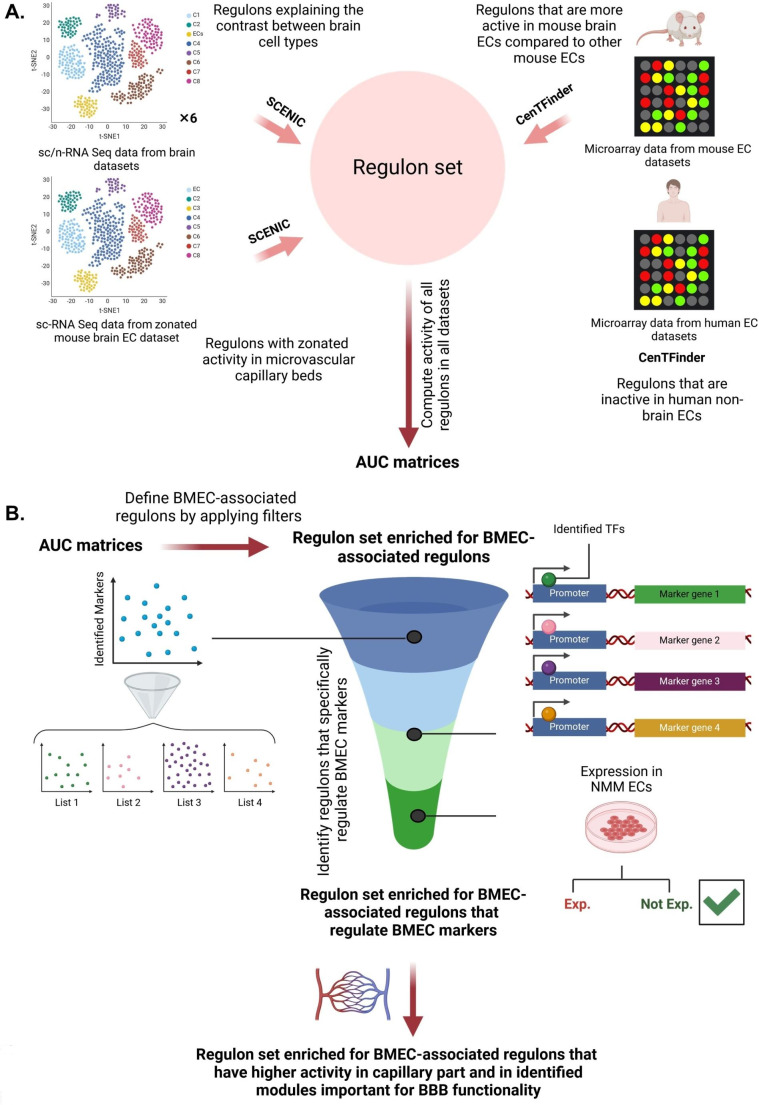
An overview of the transcriptomic analysis to identify the candidate master regulators in brain ECs.**(A)** The brain EC regulons were determined using seven brain sc/snRNA-Seq datasets (by employing SCENIC pipeline) as well as murine microarray datasets from ECs (by employing CenTFinder pipeline). Next, the activity of all identified regulons (AUC matrices) was computed using the expression rankings from brain EC populations of sc/snRNA-Seq datasets, murine microarray datasets of ECs, zonated murine brain EC dataset, and non-brain EC microarray datasets to specify the regulons with high activity in brain ECs and in capillary bed ECs, and inactive in non-brain ECs. **(B)** Schematic representation of the approach employed for narrowing down the list of transcription factors (TFs). All identified candidate TFs were first filtered based on their potential to regulate four sets of brain EC gene marker expression. We then further prioritized the TFs based on their low (/lack of) expression in NMM-iETV2 ECs, their activity in microvascular capillary beds as well as the mutual information (MI) between the vascular bed position and the GRN activity, their enrichment in important identified modules from cenTFinder analysis, and their expression in fetal brain ECs (created by Biorender.com). BMEC: Brain microvascular endothelial cells

To identify candidate TFs that might fate hPSC-ECs to a BMEC fate, we used a multipronged in silico approach, available at the time of analysis, which included four individual datasets (Fig. 2A). Several murine microarray datasets were used to derive the regulons that explain cell type differences between the brain ECs and other ECs. Human microarray data of *non-brain* EC datasets [26] were used to determine the inactive regulons. Multiple sc/snRNA-Seq studies, published in the literature [38–40, 42–44], were analyzed to identify the regulons that specify the contrast between different brain cell type (including brain ECs). Finally, a zonated brain EC dataset was used to identify the regulons with zonated activity in BMECs [41].

In a first analysis, we compared the transcriptional signature of murine brain ECs with ECs from other organs available from microarray studies (including 193 datasets of samples) (www.ebi.ac.uk/arrayexpress) (Supplementary Fig. 2A). Differential expression analysis between brain ECs and all other ECs identified a number of confirmed brain EC markers (such as Ocln, Abcb1, Itm2a, Itih5, Vwf, and Vtn) [53] as well as several putative additional markers (Supplementary Fig. 2B). To identify TFs that might fate ECs to brain ECs, we first defined the co-expressed genes using the gene co-expression analysis (i.e. WGCNA) [54] and identified 18 unique transcriptional modules (i.e. clusters of highly correlated genes) (Supplementary Fig. 2C). GO analysis confirmed the presence of several BMEC associated modules (e.g. Pink module, Supplementary Fig. 2E). All the modules were then renamed based on their enriched GO terms, for instance the pink module was renamed “the import module” (Supplementary Fig. 2D). GSVA [55] identified the higher activity of modules such as “*cytoskeleton*, *focal adhesions*, and *cell-substrate junctions*”, “*Junctions*” and “*import*” in brain ECs compared to other-ECs (Supplementary Fig. 2F). Finally, to detect candidate TFs involved in each module, RcisTarget analysis [37] was used to analyze the enrichment of TF binding motifs in the cis-regulatory regions of non-TF genes. This identified TFs such as *Erg*, *Etv4*, *Ets1*, *Tfap2a*, *Churc1*, *Cux1*, *Elf1*, *Tfap2c*, and *Etv6* as regulators of the “import” module with a cluster size of 478 genes (Supplementary Fig. 2G). Additionally, assessing the expression of these TFs in the scRNA-Seq dataset of sorted ECs in fetal brain [56] demonstrated the expression of these TFs (Supplementary Fig. 3A).

Next, we assessed sc/snRNA-Seq data of human and mouse brains, also including a zonated mouse brain EC dataset to further define the transcriptome of brain ECs (Fig. 2A) [38–44]. Using SCENIC, regulons were identified in each study. Subsequently, the activity of these regulons together with the previously identified regulons from the microarray data (Fig. 2A), was computed using the *AUCell* package [37].

**Fig. 3:**
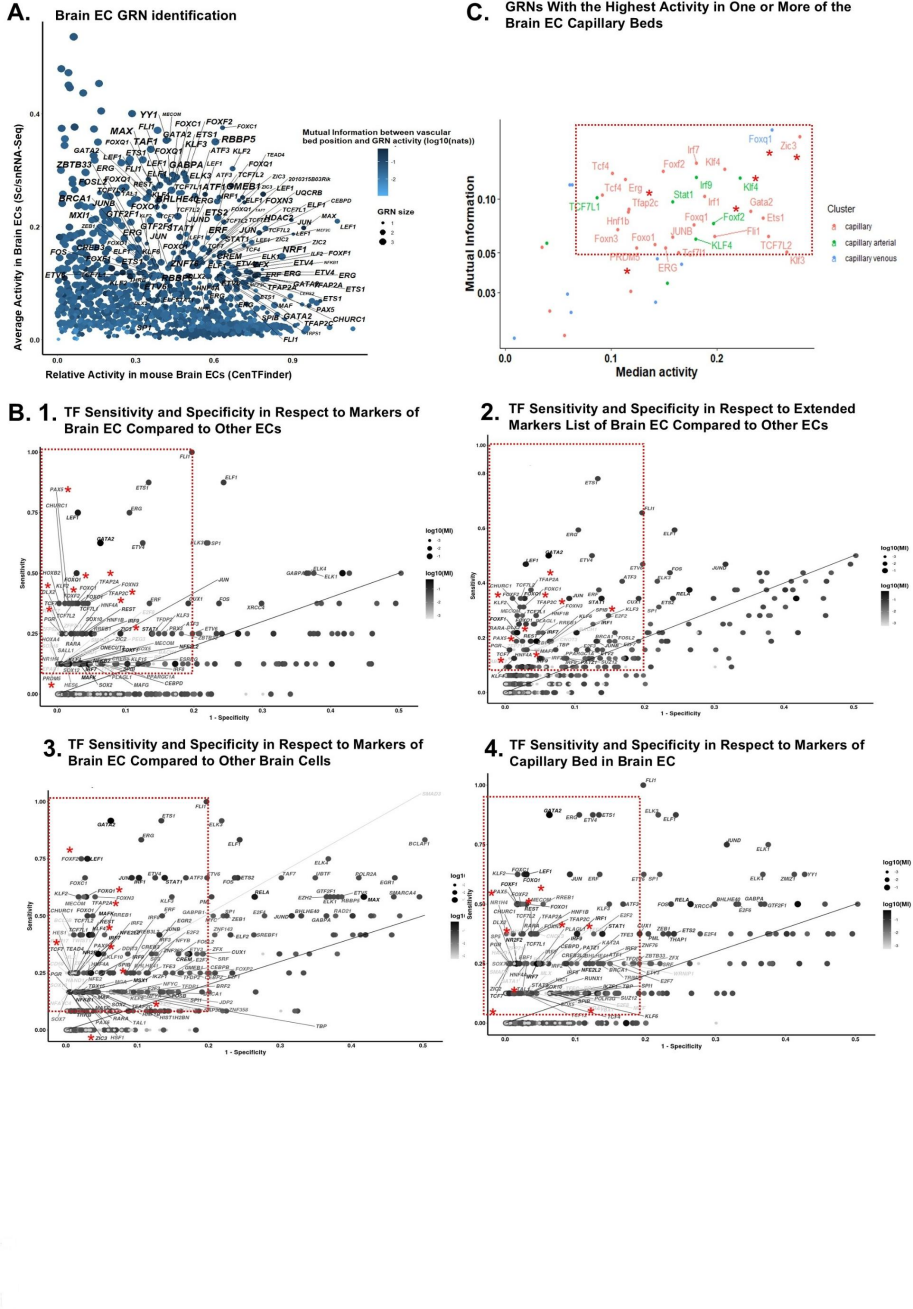
Identification of putative master TFs to fate hPSC-derived ECs towards BMEC-like cells.**(A)** Initial list of identified TFs based on their average activity in ECs from sc/snRNA-Seq studies of brain and their relative activity in mouse brain ECs compared to non-brain ECs. The mutual information (MI) between the vascular bed position (capillary part) and the GRN activity is shown through the scaled bar colors and dot sizes. **(B)** Identified TFs with high sensitivity and specificity for detecting marker genes in brain EC population from sc/snRNA-Seq datasets that possess at least four-fold **(1)** or two-fold change **(2)** expression in murine brain ECs compared to other ECs, or the marker genes with at least four-fold change expression in the brain EC population of the sc/snRNA-Seq datasets compared to other cells in the sc/snRNA-Seq studies **3)**, and marker genes in the brain EC population from sc/snRNA-Seq datasets that were at least two-fold differentially expressed in brain ECs compared to other ECs and were expressed higher (logFC > 0) in capillary beds compared to other large vessels **4)**. TFs with high sensitivity (> 0.1) and specificity (> 0.8) for all the considered marker genes were selected for the next round of screening (shown inside the red dotted box). The final selected candidates are labelled with red asterisks with respect to each marker gene set. **C**. Top TFs (from all datasets) with the highest activity (Median AUC > 0.08) in capillary veins or arteries of murine brain ECs (MI between the vascular bed position (capillary part) and the GRN activity > 0.05). The final selected candidate TFs are labelled with red asterisks. TF genes identified in human or mouse datasets are depicted with upper or lower case, respectively)

The AUC values was calculated utilizing *four* gene-expression rankings. This was done by employing the expression matrices from (1) EC clusters in three sc/snRNA-Seq studies with highest gene coverage (i.e. Allen human [40] and the mouse [42] Brain Map datasets as well as the study by Fan et al. [43] (Supplementary Fig. 4A)), (2) murine ECs, and (3) human non-brain ECs from microarray datasets. However, the ranking based on non-brain ECs was reversed as we were interested in regulons that are underrepresented in this dataset. The calculated AUC values from each dataset were then used to compute the *median activity* of regulons in “brain ECs from sc/snRNA-Seq studies” and “human non-brain ECs from the microarray datasets”. Also, we computed the *relative activity* in brain ECs versus other ECs based on the calculated AUC values in murine dataset from microarray data. Finally, we used the gene expression matrix of ECs with specified subtypes (including the capillary part) for building the fourth expression ranking and calculation of regulon’s activity [41]. Overall, this resulted in the identification of 986 candidate TFs (Fig. 3A).

To narrow down the list of candidate TFs, a series of downstream analysis was performed (Fig. 2B). First, the list of TFs was narrowed down based on the potential of TFs to regulate important marker genes. These marker genes were determined from the identified putative markers from all datasets (supplementary Fig. 4B) based on: (1) their expression in brain ECs versus other ECs, (2) their expressions in the EC cluster of the three studies with the highest gene coverage (i.e. Allen human [40] and mouse [42] Brain Map, and Fan et al. [43]), (3) their expression in capillary bed brain ECs compared to larger vessels brain ECs, and (4) their lack of expression in non-brain ECs. Eight and 32 marker genes were determined respectively when the identified marker genes in Allen and Fan et al. datasets were ranked based on at least four or two-fold change expression in murine brain ECs compared to other ECs and the 90th percentile expression of less than 7 and 7.5-fold change in human non-brain ECs compared to brain ECs (e.g. *SLCO1A2*, *ITIH5*, *PROM1*, *VWA1*, *SLC19A3*, *CA4*, *ELOVL7*, *APCDD1*) (supplementary Fig. 4C). Twelve marker genes were identified when the marker genes were ranked based on at least four-fold change expression in brain ECs from the Allen and Fan et al. datasets compared to other cells in sc/snRNA-Seq studies (*i.e. CLDN5*, *ITM2A*, *FLT1*, *BSG*, *ID3*, *SLC2A1*, *IFITM3*, *ENG*, *ID1*, *GNG11*, *EBF1*, *ABCB1*) (supplementary Fig. 4C). Additionally, ranking the identified marker genes in the Allen and Fan et al. datasets based on at least two-fold change expression in brain ECs compared to other ECs, the 90th percentile expression of less than 7.5 fold change in human non-brain ECs compared to brain ECs, and higher expression (logFC > 0) in capillary beds compared to other large vessels (supplementary Fig. 4D), identified eight marker genes, *i.e. SLC38A3*, *CA4*, *PROM1*, *VWA1*, *SLCO1A2*, *LEF1*, *FOXQ1* and *LMO2* (Top 40 markers of capillary brain ECs in comparison with other large vessels is also shown in the supplementary Fig. 4D).These markers were then used to further narrow down the TF list by assessing the TF sensitivity (i.e. by determining the fraction of markers that are regulated by a given TF) and specificity (by determining TFs ability to only regulate brain EC markers) (Fig. 3B). By selecting the TFs with higher sensitivity than 0.1, higher specificity than 0.8 and MI score more than − 1.3, 81 TFs were identified (Supplementary Table 1).

The expression of all 81 candidate TFs was then evaluated in NMM-iETV2 ECs (which received no doxycycline from day 6 onwards) and only the TFs with TPM expression value < 2 were selected. This narrowed down the number of TF candidates to 24 (we also retained *ZIC3* because of its potential for inducing a brain EC fate [2]) (Supplementary Table 2). Subsequently, we evaluated the median activity of these candidate TFs in capillary bed ECs; the mutual information (MI) between the vascular bed position and the regulon’s activity (Fig. 3C) [41]; there presence in important modules from the cenTFinder analysis (Supplementary Fig. 2G) (*e.g. import*, *junctions*, and *lipid metabolism and membrane transport modules*); and their expression in proliferating stem cells differentiating to ECs in fetal CNS based on Wälchli et al. [56], (Supplementary Fig. 3B). This resulted in the final selection of the following 12 candidate TFs: *ZIC3*, *FOXF2*, *FOXQ1*, *TCF7*, *KLF4*, *PRDM5*, *TFAP2A*, *TFAP2C*, *SPIB*, *DLX2*, *PAX5* and *HNF4A*.

### Single Cell Transcriptome Analysis Revealed the Similarity Between a Proportion of Transduced Cells and the Developing Human Brain ECs

To test which TF or TF combination can direct hPSC-ECs to a BMEC-like phenotype, we prepared a library of TF encoding lentiviral vectors. cDNA of the 12 TFs were cloned in a viral vector backbone together with a HA tag at the 5’ site of TF ORF and a unique 20-bp barcode, 196-bp upstream of the 3’-LTR region (Supplementary Fig. 5A). Vectors were transfected individually in HEK-293T cells, to demonstrate protein expression of all TFs based on HA immune fluorescence staining (Supplementary Fig. 5B). Day 6 NMM-iETV2 ECs were then transduced with the 12-candidate TF library and after four days culture without doxycycline, transduced cells underwent scRNA-Seq (Supplementary Fig. 5C).

**Fig. 4:**
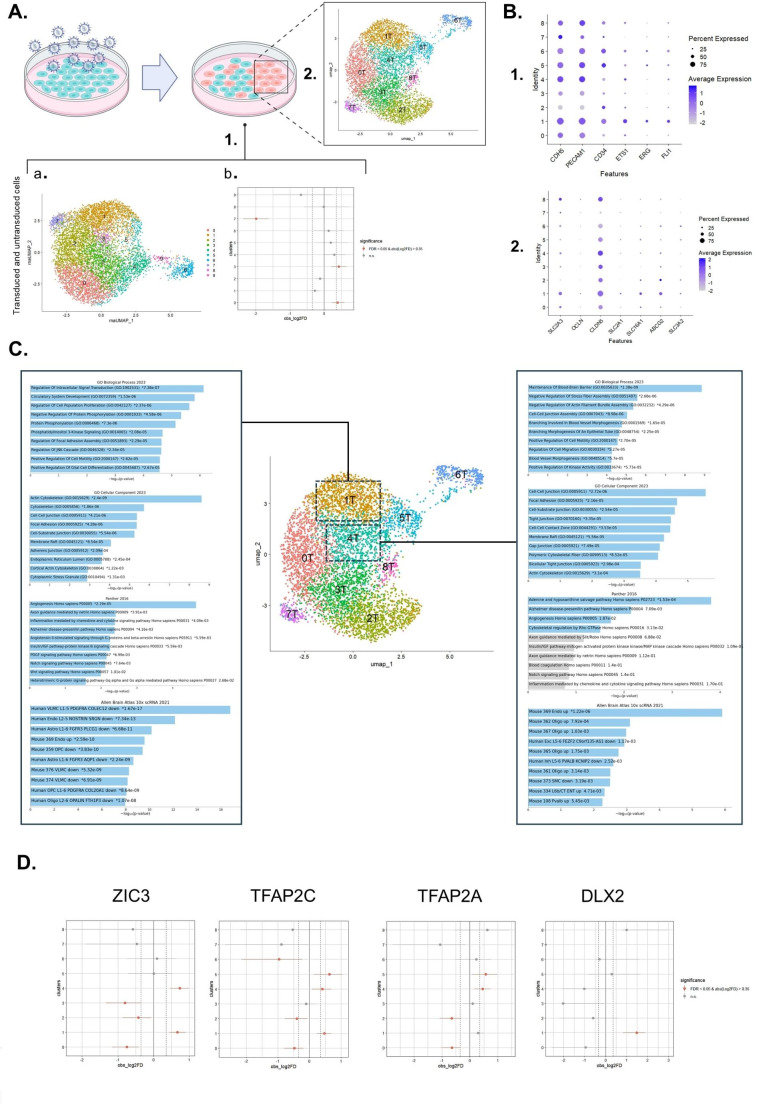
Single-cell RNA sequencing results revealed the transcriptomic similarity between brain ECs and transduced cells in cluster 1^T^ and 4^T^. **(A)** The Uniform Manifold Approximation and Projection **(**UMAP) embedding of: 1a) transduced and untransduced as well as 2) only transduced ECs, each specified 10 and 9 cell clusters, respectively. 1b) Point-range plot indicating that untransduced cells are mostly distributed in cluster 7, while transduced cells are mostly enriched in clusters 0 and 3. **(B)** The average expression and percentage of expressing cell for: (1) the EC lineage markers and (2) brain EC markers per cell cluster of the transduced cells. **C.** Enrichment analysis results of clusters 1^T^ and 4^T^ demonstrating the enriched pathways (Panther 2016 database), gene ontology (GO) annotations (including biological process (BP) and cellular component (CC) annotations), as well as similar cell types in brain according to the Allen brain atlas 10X scRNA sequencing results 2021 from human and mouse brain regions using EnrichhR (https://maayanlab.cloud/Enrichr/). **D.** Point-range plot indicating the significantly enriched TFs in clusters 1^T^ and 4^T^

The Seurat computational pipeline was used to cluster the cells according to their gene expression profiles. UMAP dimensionality reduction identified 10 cell clusters whereby cluster 7 contained the highest number of untransduced cells and clusters 0 and 3 contained the highest number of transduced cells, based on the presence of TF barcodes (Fig. 4A.1). As we were interested in the effect of TFs on cell identity, cells with barcodes were separated and re-clustered. The UMAP visualization of the 9 identified clusters is shown in Fig. 4A.2.

To assess the putative identity of each cluster from the transduced cells, we first determined the expression of known EC markers (such as *PECAM1*,* CD34*,* CDH5*,* ETS1*,* FLI*, and *ERG*) and brain EC markers (such as *CLDN5*, *OCLN*,* SLC2A1*,* SLC2A3*,* SLC3A2*,* ABCG2*,* SLC16A1*) in each cluster (Fig. 4B). This demonstrated, not unexpectedly, that most of the cells, specifically in clusters 0^T^, 1^T^, 4^T^, 5^T^, 6^T^, 7^T^, and 8^T^ expressed high levels of EC-specific markers (Fig. 4B.1). Additionally, the tight junction protein *CLDN5* was shown to be highly expressed in clusters 1^T^, 4^T^. Of note, cells in cluster 1^T^ expressed a high level of glucose transporter transcripts (i.e. *SLC2A3*, and to a lesser degree *SLC2A1*), the monocarboxylate transporter *SLC16A1*, the transporter *ABCG1*, and the amino acid transporter *SLC3A2* (Fig. 4B.2). Subsequently, functional enrichment analysis of differentially higher expressed genes in each cluster was used to identify the candidate cluster(s) with the greatest similarity with BMECs. Gene Ontology (GO) analysis demonstrated that clusters 1^T^ and 4^T^ had high resemblance to BMECs (Fig. 4C and Supplementary Fig. 6A). Specifically, the terms “Maintenance of Blood-Brain Barrier (GO:0035633)” and “Blood Vessel Morphogenesis (GO:0048514)” were among the highest enriched terms in GO biological processes (BP) analysis in cluster 4^T^. “Cell- Cell Junction (GO:0005911)” and “Focal Adhesion (GO:0005925)” terms were significantly enriched in both clusters 1^T^ and 4^T^ in GO cellular component (CC) analysis. The “Angiogenesis Homo sapiens (P0005)” pathway was also shown to be enriched in cluster 1^T^ according to the Panther 2016 database (Fig. 4C). When we used the Allen Brain Atlas 10X SCRNA 2021 database, we found that both clusters 1^T^ and 4^T^ displayed a gene expression profile similar to murine brain ECs and the term “Human Endo L2-5 NOSTRIN SRGN down” was enriched in cluster 1^T^ (Fig. 4C). We also identified a distinct gene signature related to Wnt pathway signaling in cluster 1^T^ (consisting of among others *SMAD1*, *TCF7L2*, *TCF7L1*, *CTBP2*, *CDH2*, *GNAQ*, *ITPR2* and *ARID1A*) (Supplementary Fig. 6B). Genotyping each cell based on the unique TF-barcode(s) present, revealed that the TFs *ZIC3*,* TFAP2C*,* DLX2*, and *TFAP2A* were significantly more frequent in clusters 1^T^ and 4^T^ (Fig. 4D). Furthermore, we assessed if cells had been transduced simultaneously with a combination of 2 or 3 TFs. Several double transduced cells were identified to have the final TF candidates. Assessing the expression of the BMEC marker genes *OCLN*, *SLC2A1* and *SLC2A3* in cells with at least one of the four candidate TFs revealed that cells with ZIC3 barcodes show a higher expression of all markers especially in clusters 1^T^, 4^T^, and 6^T^ (Supplementary Fig. 7). In addition, the double transduced cells with any combination of the candidate TFs demonstrated to have increased expression of the marker genes especially in clusters 1^T^ and 4^T^ (Supplementary Fig. 7). This suggests a possible causal effect of TF presence on BMEC-like gene expression profile.

**Fig. 5:**
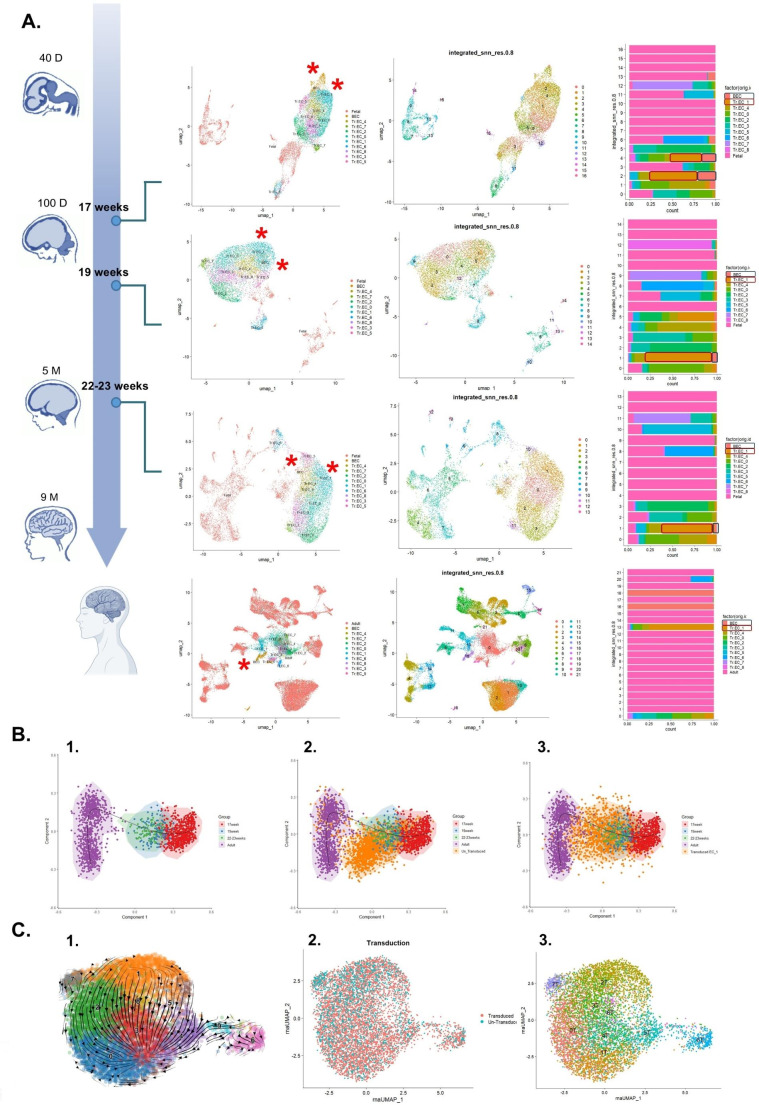
A subset of transduced ECs is similar toin vivodeveloping brain ECs. **(A)** UMAP representation of integrated human in vitro transduced ECs and in vivo brain cells in a chronological order revealed the similarity between the transcriptomic profile of 1^T^ and 4^T^ (to a lesser degree) to the developing brain ECs (labeled with red asterisks in related UMAPs). The stacked bar plots show the proportion of in vivo brain ECs (i.e. BEC) and transduced EC clusters (e.g. Tr_EC_1) in each integrated cluster. BEC: Brain ECs. (partially created by Biorender.com) **(B)** Trajectory inference analysis of **1)**in vivo brain ECs confirmed the chronological order of developing brain ECs1Positioning the coordinates of **2)** untransduced cells and **3)** cluster 1^T^ on the trajectory visualization. **(C) (1)** The results of velocity projections onto the UMAP embedding for the mixed population of transduced and untransduced cells identified the directed trajectory of cells from a more untransduced status toward more transduced cells **(2)** and to the putative more BMEC-like state in cluster 1^T^**3)**. (The location of transduced clusters are positioned in the UMAP plot of the mixed population of transduced and untransduced cells)

To assess the similarity of the cells in clusters 1^T^ and 4^T^ with brain-ECs at different stages of brain development, we integrated the scRNA-Seq data of the transduced hPSC-ECs with a series of fetal and adult brain datasets [43, 47, 48] (Fig. 5A, Supplementary Figs. 8, 9). We also used datasets from in vitro cultured primary human BMECs [22], day 11 induced (i)BMECs (based on [16, 22], rECs (reprogrammed Epi-iBMECs with *ETV2*, *ERG*, and *FLI1*) [22] (Supplementary Fig. 10D, E), and human fetal liver [49] (Supplementary Fig. 10A-C), to assess their similarity to the transduced ECs. We observed that clusters 1^T^ and 4^T^ have a similar expression profile with fetal brain ECs in an age-dependent manner, which was confirmed by the trajectory analysis of in vivo brain ECs and cluster 1^T^ or untransduced cells (Fig. 5A, B and Supplementary Fig. 8A-D, and 9A-B). Specifically, fetal BMECs from a 19 week-old fetus displayed the highest similarity with cluster 1^T^ of the transduced ECs. Of note, a subset of untransduced cells appeared to have a transcription profile similar to younger fetal brain ECs, which may be the result of spontaneous unguided differentiation (Fig. 5B). Subsequently, we defined which barcodes were mostly present in clusters integrated with fetal brain ECs. This also identified the barcodes for the *ZIC3*, *TFAP2C*, *TFAP2A*, and *DLX2* TFs (Supplementary Figs. 8E, F, and 9C). By contrast, integration of scRNA-Seq data with liver ECs, revealed that clusters 2^T^ and 6^T^ were more similar to a cluster of fetal liver ECs (Supplementary Fig. 10A-C). Cells in 2^T^ were enriched for *FOXF2* barcodes (Fig. 4D). Finally, transduced cells in cluster 1^T^ possess an expression profile more similar to developing brain ECs compared to iBMECs and cultured BMECs (Supplementary Fig. 10D), while a small proportion of rECs co-clustered with cells in cluster 1^T^ and brain ECs from 19 week old fetus (Supplementary Fig. 10D, E).

Finally, we used the combination of transduced and untransduced cells to perform RNA velocity analysis (Fig. 5C.1, Supplementary Fig. 10F, G). Interestingly, clusters containing most untransduced cells were the likely start site in the trajectory while clusters with most transduced cells were the final destination clusters (Fig. 5C.2). By tracing back the transduced EC clusters on the resulting UMAP visualization, we determined that the final destination of cell fate was towards cluster 1^T^ and for many cells this was achieved by passing through cluster 4^T^ (Fig. 5C.3). Moreover, based on the velocity length, cells differentiated faster in cluster 4^T^ compared to other clusters (Supplementary Fig. 10F). Based on these results, we concluded that a combination of *ZIC3*, *TFAP2C*, *TFAP2A*, and *DLX2* TFs might be required and possibly sufficient to create BMEC-like cells from hPSC-ECs.

## Discussion

The BBB serves as a highly selective border through which small hydrophobic molecules can diffuse into the brain, whereas nutrients are actively transported through the BBB to support neural function [57]. The BBB also protects the brain from toxic molecules and a wide range of therapeutics and limits trafficking of immune cells. Disruptions in components of the BBB or NVU can lead to neurological and pathological conditions [58]. For instance, vascular dysfunction and BBB disruption are common features of neurodegenerative diseases such as Alzheimer’s Disease, Parkinson’s Disease, Amyotrophic Lateral Sclerosis, and Huntington’s Disease, and may contribute to their onset and progression [59]. Therefore, the BBB is regarded as a potential therapeutic target in neurodegenerative disorders and BBB models encompassing cells from patients with neurodegeneration could be used for developing therapeutic strategies targeting BBB integrity in neurodegeneration.

Although some studies have been published describing the generation of BMEC like cells from hPSCs [15–19], these cells do not appear to have all salient features of ECs [22]. For these reasons, we here describe the creation of immature endothelial cells by inducing over-expression of the TF *ETV2* [26], which then served as a cell platform to identify TFs that might fate such immature ECs to cells with BMEC features. We performed a very extensive in silico analysis of online available micro-array and sc/snRNA-Seq studies of brain ECs versus ECs from different tissues as well as different brain cells. This identified 12 candidate TFs that might be important for BMEC fating. A lentiviral library consist of the 12 TFs each linked to a unique barcode was used to transduce the ETV2-ECs, and scRNA-Seq was performed which led to the identification of four TFs, i.e. *ZIC3*, *TFAP2C*, *TFAP2A*, and *DLX2*, that fated immature ECs to cells transcriptionally similar to mid-gestational human brain ECs.

To ensure that the hPSC-derived BMEC-like cells would have a definite EC phenotype [22], we created ECs cultured in NMM, also compatible with astrocytes that are an integral part of the BBB, through overexpression of the EC-master TF *ETV2*. *ETV2* is expressed in ECs early during development (E7.0-E9.5 in mice [52]) but it’s expression levels are minimal in human fetal brain ECs (Supplementary Fig. 1B-F). Consistent with the notion that ETV2 is no longer required to maintain an EC fate, once fully established, removal of doxycycline to prevent further induction of *ETV2* did not affect the EC phenotype, characterized by the persistent expression of the EC-specific TFs (*ETS1*,* FLI1*, and *ERG*) and EC-specific cell markers, as well as the preserved ability of the ECs to make vascular tubes.

GRNs have a central role in defining the cell identity. Since the identification that MYOD is sufficient to convert a fibroblast to a skeletal muscle cell [60], innumerous studies have exploited the use of overexpression of core regulatory TFs to induce cell fate through cellular (re)programming [61, 62]. Over the last decade, numerous methods have been described for the identification of core regulators of GRNs from transcriptomic profiles of cells with high resolution. SCENIC analysis is one example of such approaches which takes advantage of sc/snRNA-Seq data to identify (combinations of) TFs with a central role in driving cell type specific transcriptomes [37]. Additionally, our lab recently introduced the CenTFinder workflow which designed to use microarray or bulk RNA sequencing data for the identification of TFs and pathways with significant contribution in defining a certain biological state [26]. Here, we used several sc/snRNA-Seq data from adult and fetal brain of mice and human to identify core TF regulators in brain (microvascular) ECs. This was complemented with CenTFinder analysis of multiple microarray data from brain and other organ ECs. Upon merging the identified regulons from the sc/snRNA-Seq and microarray datasets, four gene-expression rankings were used to calculate the AUC value, including: (1) the expression matrix of brain ECs in three sc/snRNA-Seq datasets with the highest gene coverage, (2) the expression matrix of murine ECs from the microarray datasets, (3) the expression matrix of brain EC subtypes, and (4) the expression matrix of human non-brain ECs. The last matrix was used for a reverse ranking as we were interested in the TFs with lowest activity in non-brain ECs.

To further highlight the master regulators of brain EC development, and given the role of TFs in directing the expression of cell marker genes and hence the maintenance of cell identity [63], we assessed the identified TFs for their regulatory effect on brain EC marker genes We created a list of marker genes that were more highly expressed in brain ECs than in ECs from other organs/tissues and other brain cells, as well as marker genes that are more specific to the capillary bed of brain ECs. Among this list, we identified known brain EC marker genes that play a role in cell-adhesion (*e.g. CLDN5*, *OCLN*) [64] or transport (*e.g. ABCB1*, *SLCO1A2* [65], *Slc2a1*, *Slc38a3* [66]), and have been shown by others to be expressed in brain ECs (*e.g. ITM2A* [67], *Itih5*, *Apcdd1* [68], *ELOVL7* [69], and *Bsg* [70]. By comparing capillary bed brain ECs with larger vessels of brain, we also identified markers shown by others to be BMEC specific, such as *CA4* [71], *SLCO1A2* [72] and *LEF1* [73]. In addition, our analysis identified additional candidate BMEC marker genes such as *IFITM3*,* PROM1*,* GNG11*,* LMO2* as well as *ID1* and *ID3*.

Using this extensive list of marker genes, we narrowed down the number of candidate TFs to 81 by evaluating candidate TFs that may regulate the marker genes of brain (microvascular) ECs but not ECs from other organs or other brain cells. To further hone in on those TFs that could fate immature ECs (here ETV2-induced hPSC-ECs) to cells with BMEC features, we focused on candidate TFs not already expressed in ETV2-induced ECs and the ones that were shown to be significantly enriched in identified modules important for murine brain EC function. This resulted in a list of 12 candidate BMEC TFs, i.e. *FOXF2*, *FOXQ1*, *SPIB*, *TCF7*, *KLF4*, *PRDM5*, *TFAP2A*, *TFAP2C*, *HNF4A*, *ZIC3*, *PAX5* and *DLX2*.

Among these candidate TFs, several have previously been shown to be enriched in (embryonic) BMECs (*Foxq1* [24], *ZIC3*, *FOXF2* [74], *DLX2* [75] and *TCF7* [73]), or to have possible functional/protective roles in brain vessels (*HNF4A* [76] and *KLF4* [77]). Of note, ZIC3 has been shown to be involved in the establishment of BMEC during fetal development [74]. In addition, when overexpressed in human umbilical vein ECs (HUVECs), *FOXF2* and *ZIC3* induced expression of BMEC markers [24]). Furthermore, *DLX2* is involved in murine telencephalic angiogenesis. It is expressed in periventricular EC in E11 ventral telencephalon, partaking in regulating angiogenesis along a spatial, ventral-to-dorsal gradient [78]. *PRDM5* is known to repress transcription through recruitment of histone methyltransferase EHMT2/G9A and histone deacetylases such as HDAC1 [79]. It may affect expression of proteins involved in extracellular matrix development, maintenance and important for vascular integrity as well as molecules regulating cell adhesion [79]. *SPIB* is a member of the *ETS* transcription factor family. It is important in B-cell development, and we previously also showed that *SPI1* (which interacts with SPIB) endows iPSC-derived ETV2-ECs with expression of the scavenging receptors CD32B and MRC1 [26]. *PAX5* is another TF known to play a role in B lymphocyte development, but was also found expressed in cultured HUVECs, HMEC-1, and human pulmonary vein (HPV)EC, where it plays a role in regulating expression of endothelin-1 [80]. *TFAP2A* and *TFAP2C* are two members of the AP-2 family of TFs that play important roles in cancer development [81], but also in early embryonic development, especially of the neural crest [82] and trophectoderm [83], where they have both redundant and non-redundant functions. However, neither TF has known functions in endothelium, even if transcripts of *TFAP2A* were found in corneal endothelium differentiating from iPSC-derived neural crest [84]. There is also evidence that Wnt activates or mediates the effect of *TFAP2A* [85] and *TFAP2C* [85, 86] although this was not shown in ECs.

Four days following the transduction of these 12 TFs in ECs, we performed an extensive transcriptomic analysis on the cells and identified multiple clusters with the transcriptomic profile of ECs using scRNA-Seq. Trajectory analysis demonstrated a pattern from more untransduced cells towards more transduced cells, starting from cells with a more active translational status (clusters 2^T^, 3^T^, and 8^T^ (Supplementary Fig. 6A)), converting to cells with the biological process of “BBB maintenance” (cluster 4^T^), towards cluster 1^T^ (Fig. 5C). Cluster 1^T^ displayed a high correlation with developing BMECs, specifically with 19-week-old human fetal brain ECs. In addition, the analysis of TF-barcode(s) distribution indicated a higher frequency of the *ZIC3*,* TFAP2C*,* TFAP2A*, and *DLX2* in clusters 1^T^ and 4^T^. However, no cells were found with 3 TF barcodes. It should be noted that the frequency of some TF-barcodes was (very) low (PRDM5, HNF4A, DLX2, KLF4 and PAX5). The reason for this is not clear. When transfected in HEK293 cells, we could detect the HA tag for each of these vectors, demonstrating that the vectors encode for the TF proteins. One possibility is that the transduction efficiency of these vectors is lower than that of the other 7 TFs, and hence that they are outcompeted by the other vectors in a combinatorial transduction scheme. An alternative possibility might be that overexpression of some of these TFs might be toxic for immature PSC-ECs. Therefore, our study may not allow us to conclude that these 5 TFs play no role in BMEC fating. Nevertheless, despite the fact that relatively few cells contained the DLX2-barcode, our analysis still demonstrates that this TF may be required for creating cells with BMEC features.

## Conclusions

Overall, we suggest that *ZIC3*, *TFAP2C*, *TFAP2A*, and *DLX2* may have the potential to direct immature hPSC-ECs towards cells exhibiting mid-gestational BMEC features. However, additional studies will be required to determine which combination of these 4 TFs is necessary and sufficient to generate BMECs from hPSC; as well to demonstrate that the resulting cells have functional properties of BMECs, including transport and barrier functions. In addition, the readers are encouraged to explore the provided unfiltered list of potential TFs and apply their own criteria to further identify novel TFs as here we limited the list of final candidates to four.

## Supplementary Information

Below is the link to the electronic supplementary material.

Supplementary file1 (PDF 3 MB)

Supplementary file2 (DOCX 51 KB)

## Data Availability

The data from this study is available from the corresponding author upon reasonable request.

## Code Availability

The computational analyses were performed using R and Python, with all relevant packages appropriately cited within the manuscript.

## Abbreviations

BBB: Blood-brain barrier
BMECs: Brain microvascular endothelial cells
CNS: Central nervous system
EC: Endothelial cells
GO: Gene ontology
GRN: Gene regulatory network
GSVA: Gene set variation analysis
hESC: human Embryonic stem cell
hPSCs: human Pluripotent stem cells
LDM: Liver differentiation medium
MI: Mutual information
NES: Normalized enrichment score
NMM: Neural maintenance medium
NVU: Neurovascular unit
PC: Principal components
RT-qPCR: Reverse-transcriptase quantitative polymerase chain reaction
Sc/nRNA-Seq: single cell/nucleus RNA-Sequencing
SCENIC: Single-cell regulatory network inference and clustering
SNN: S nearest neighbors
TF: Transcription factor
TJ: Tight junction
UMAP: Uniform manifold approximation and projection
WGCNA: Weighted gene correlation network analysis
