# Supplementary figures and images for: Identification of Cell Fate Determining Transcription Factors for Generating Brain Endothelial Cells
Source: Stem Cell Rev Rep. 2025 Jan 24;21(3):744–66. doi: 10.1007/s12015-025-10842-7 (PMC11965213; doi:10.1007/s12015-025-10842-7)

**A.**

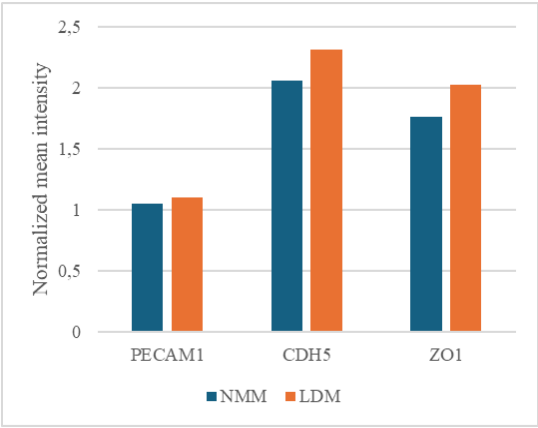

**B.**

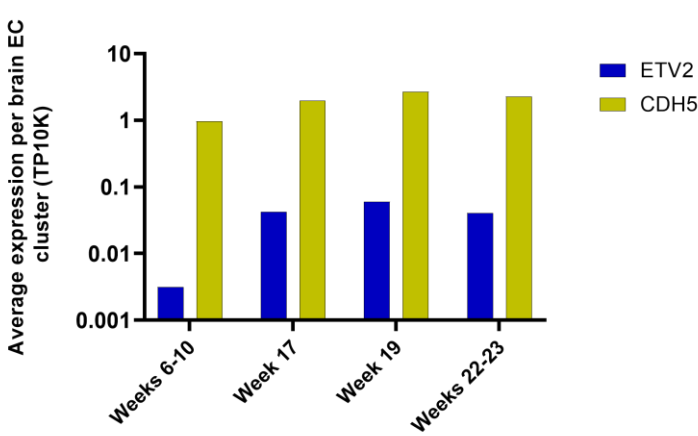

**C. Weeks 6-10**

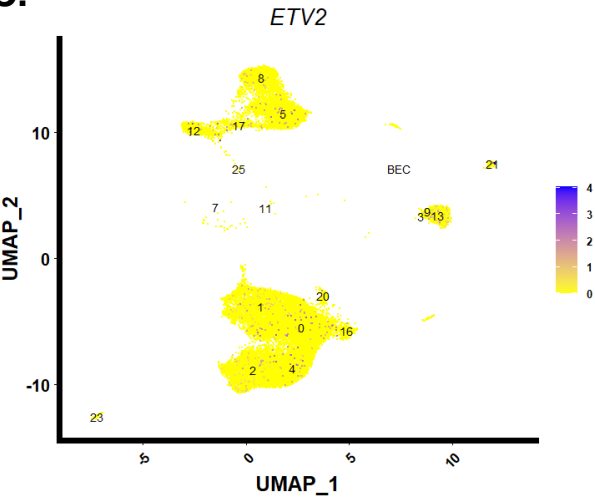

**D. Week 17**

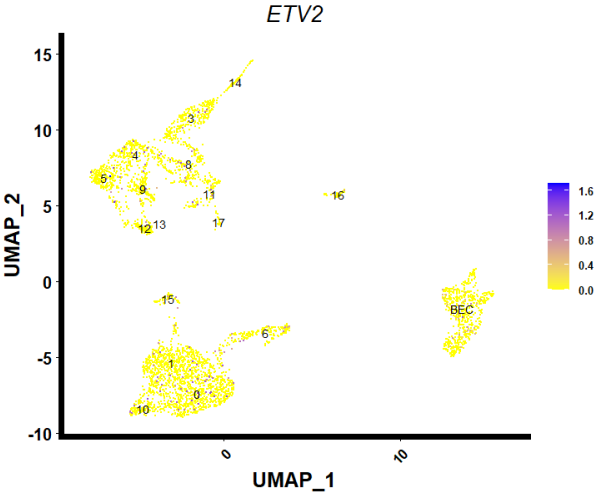

**E. Week 10**

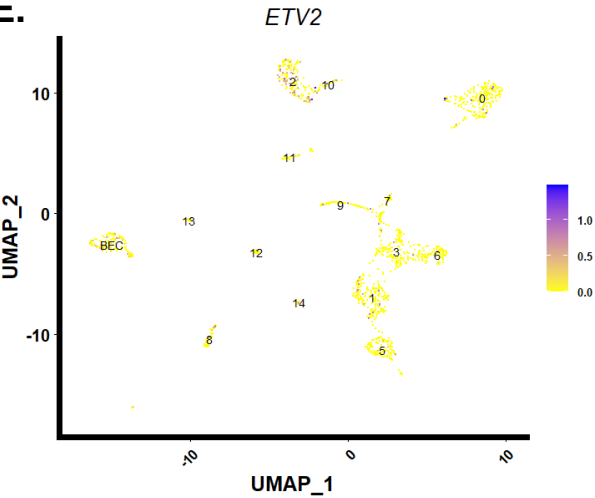

**F. Weeks 22-23**

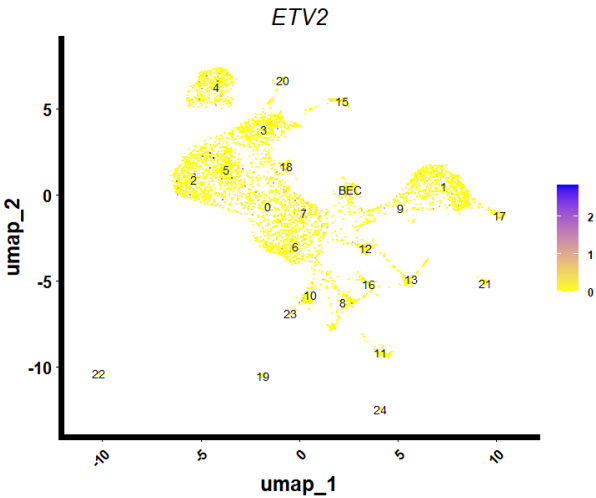

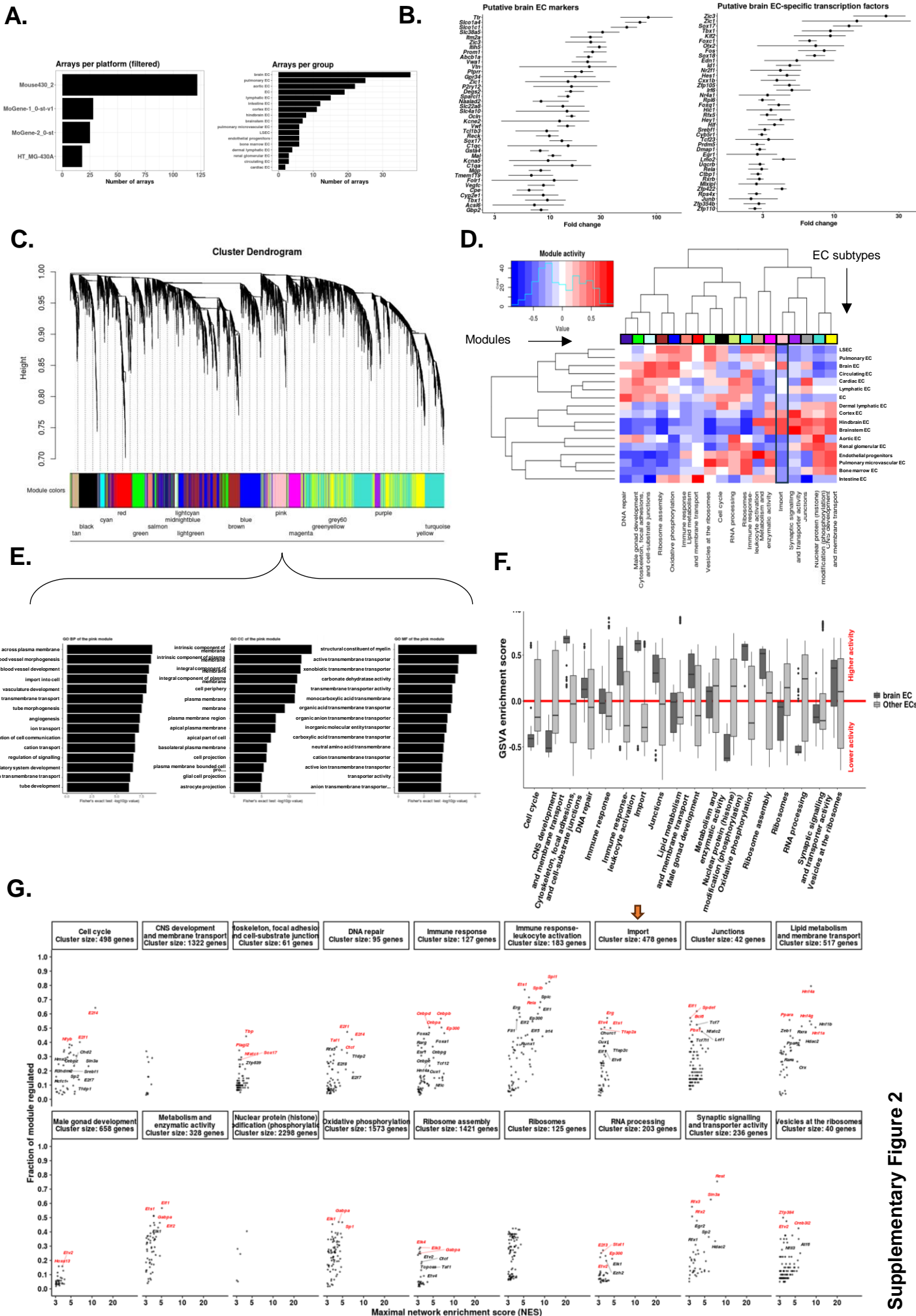

A.

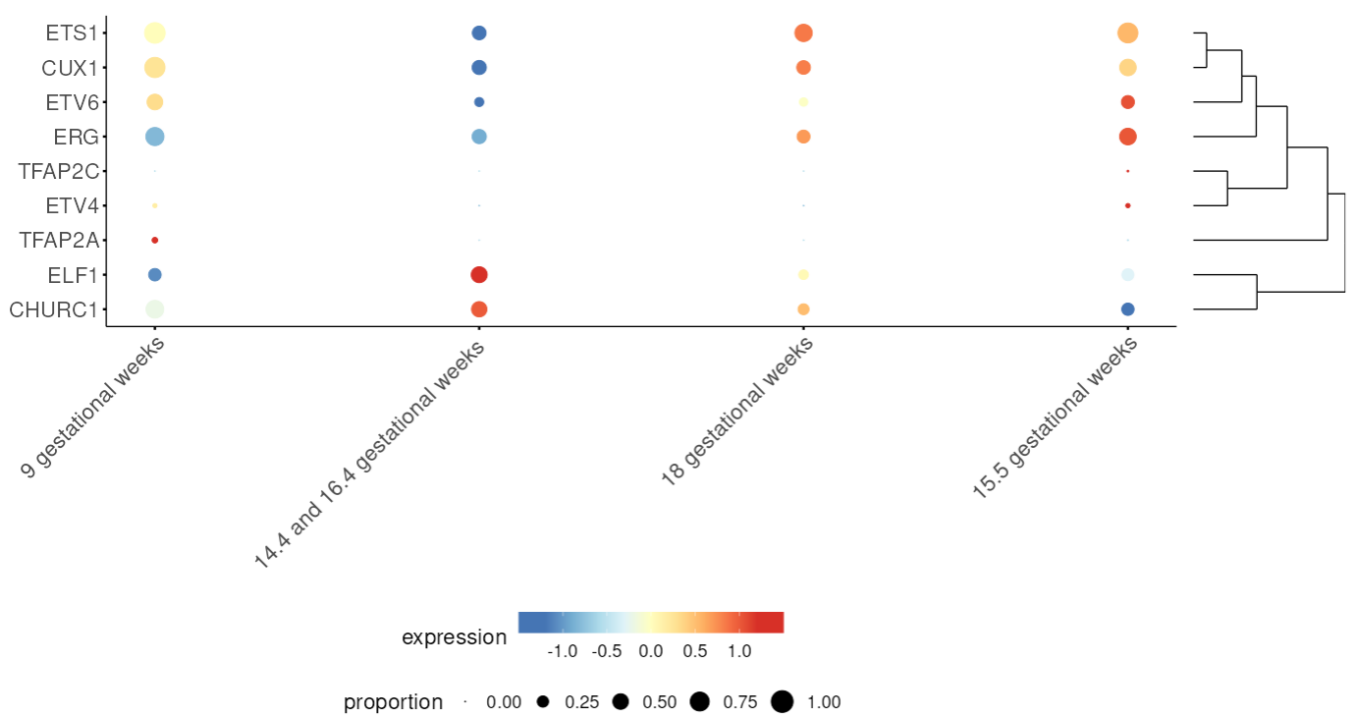

B.

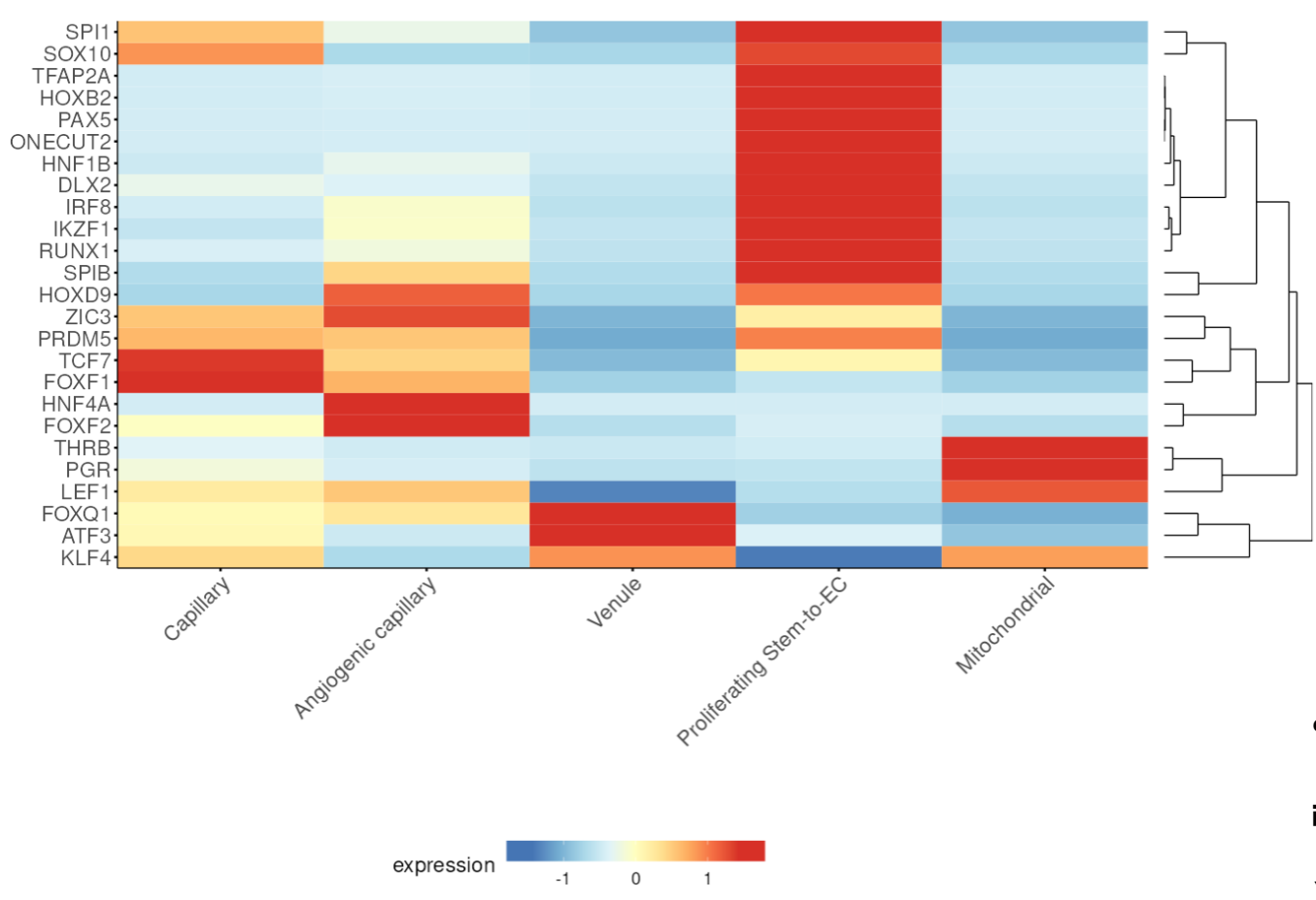

**C.**

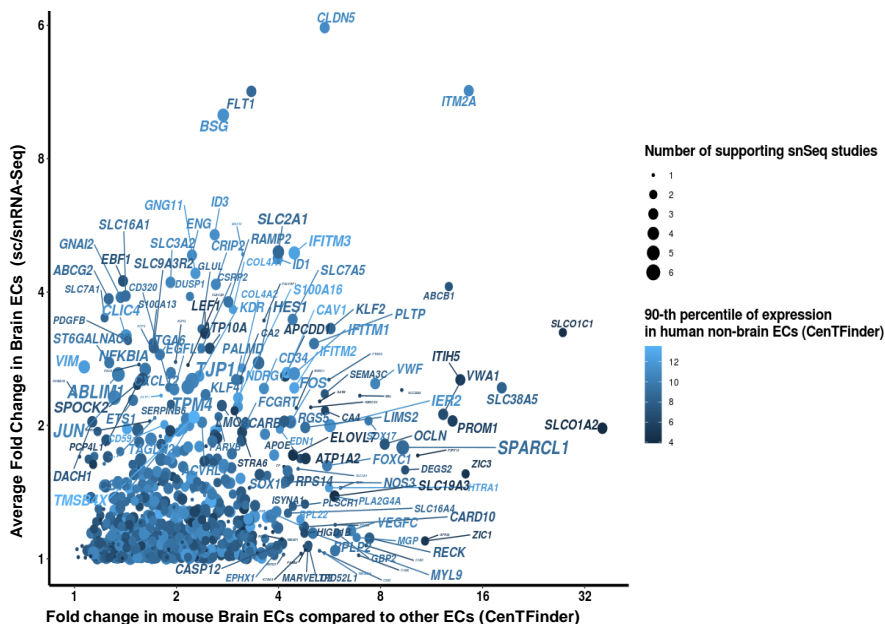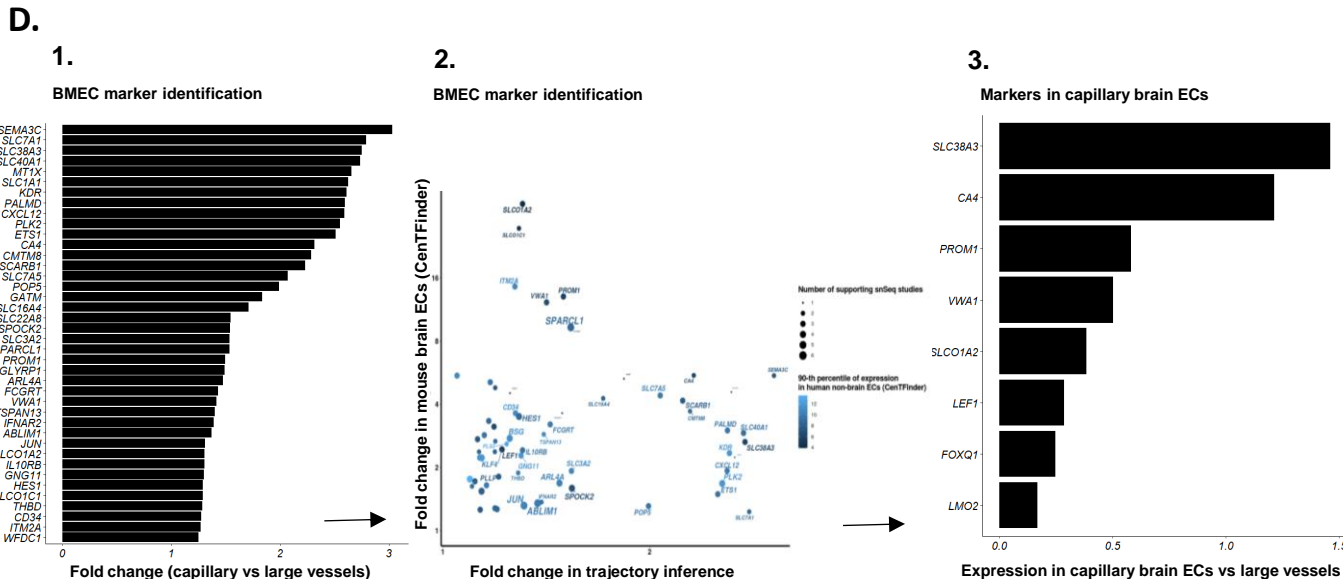

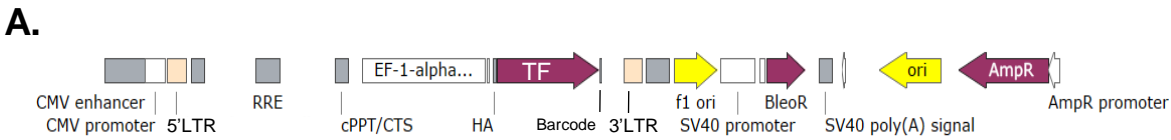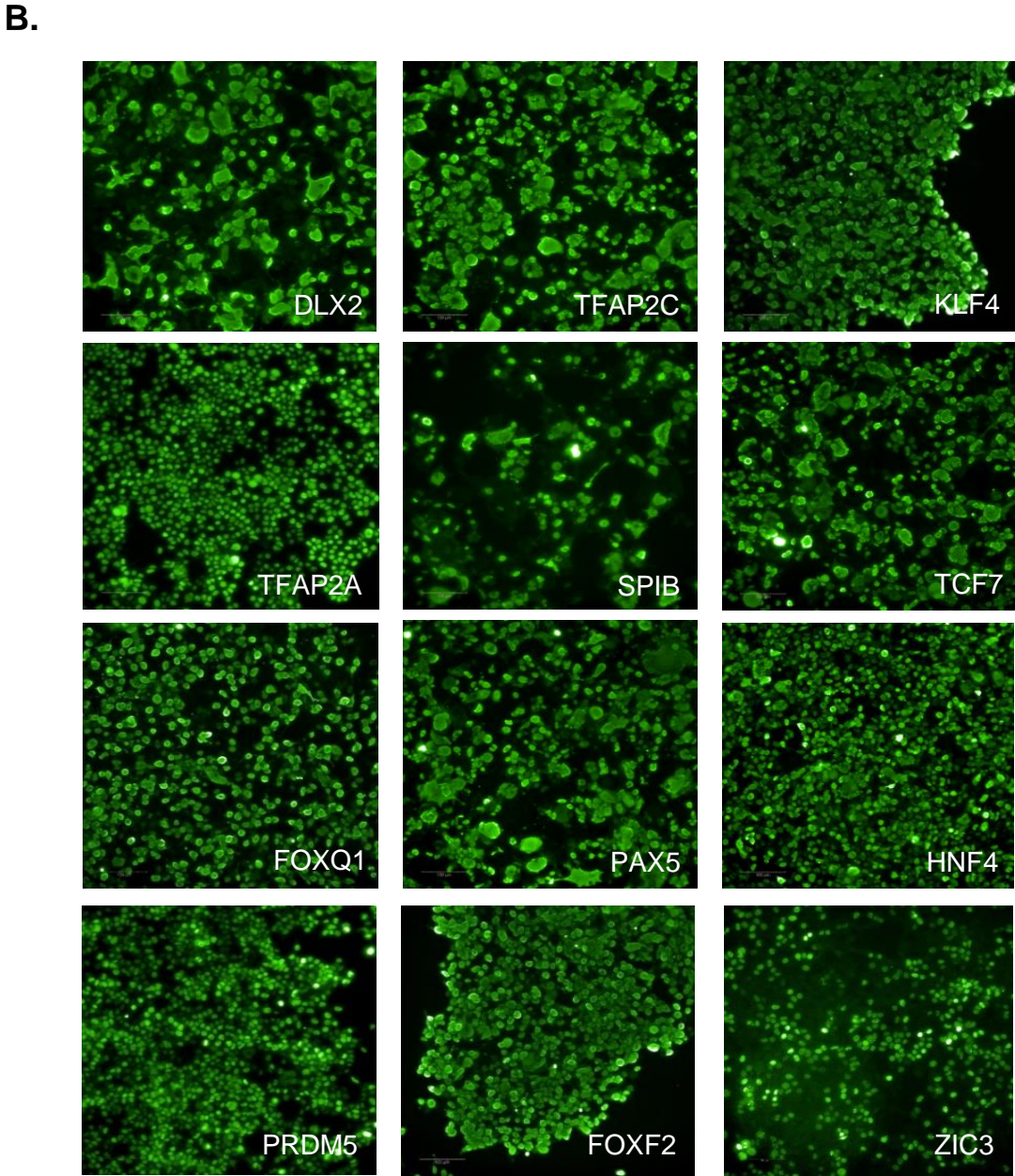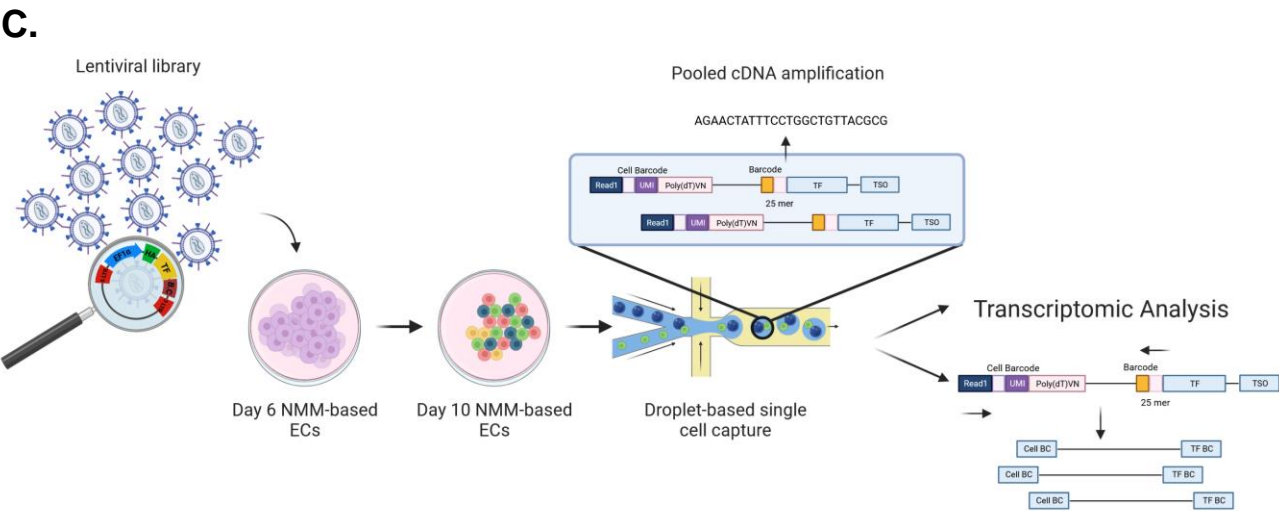

A.

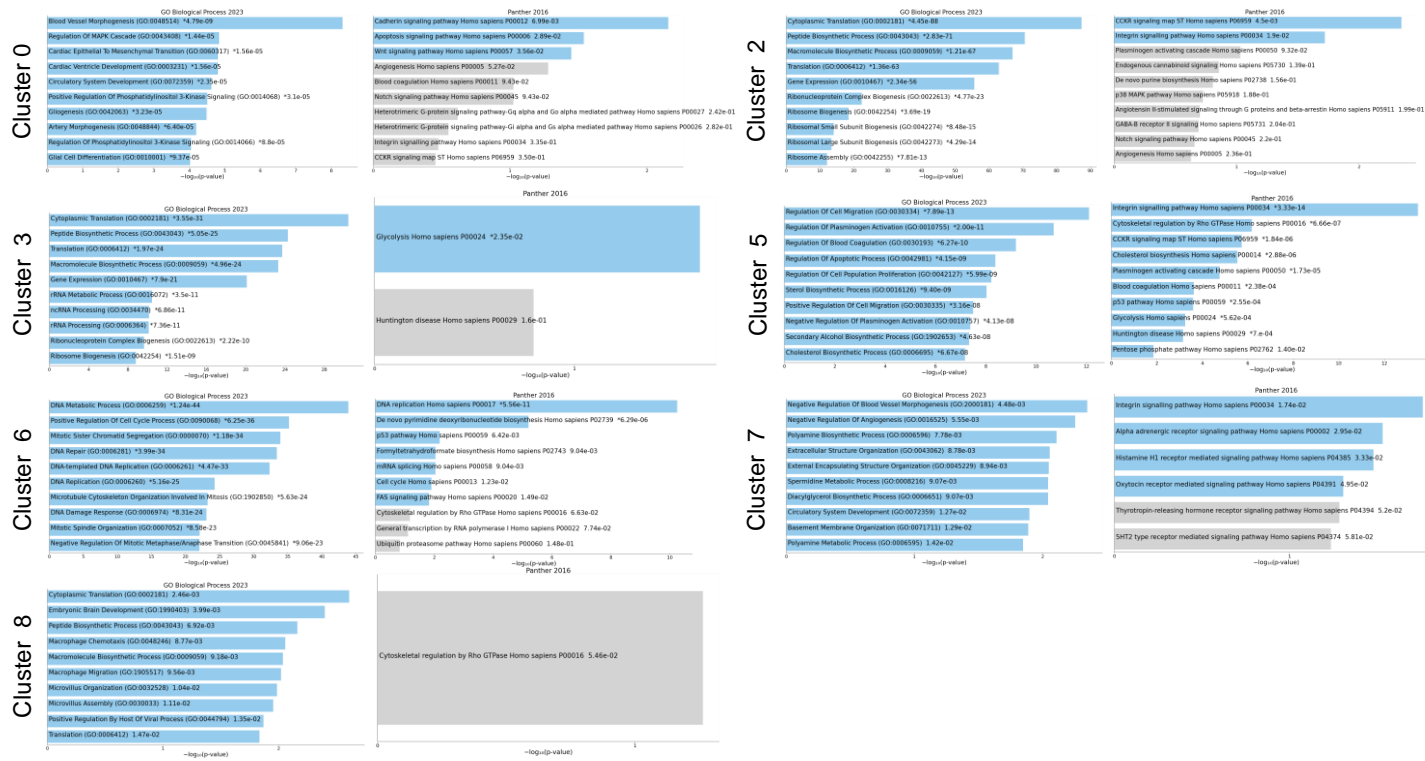

B.

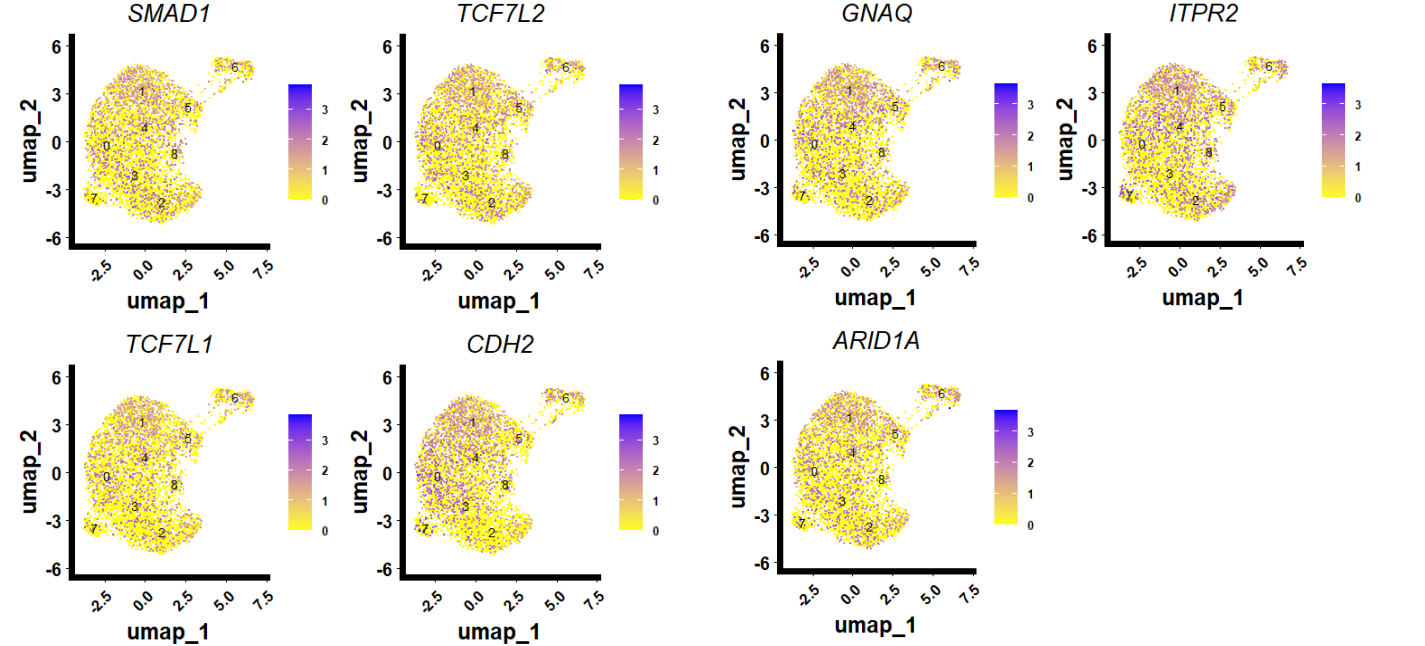

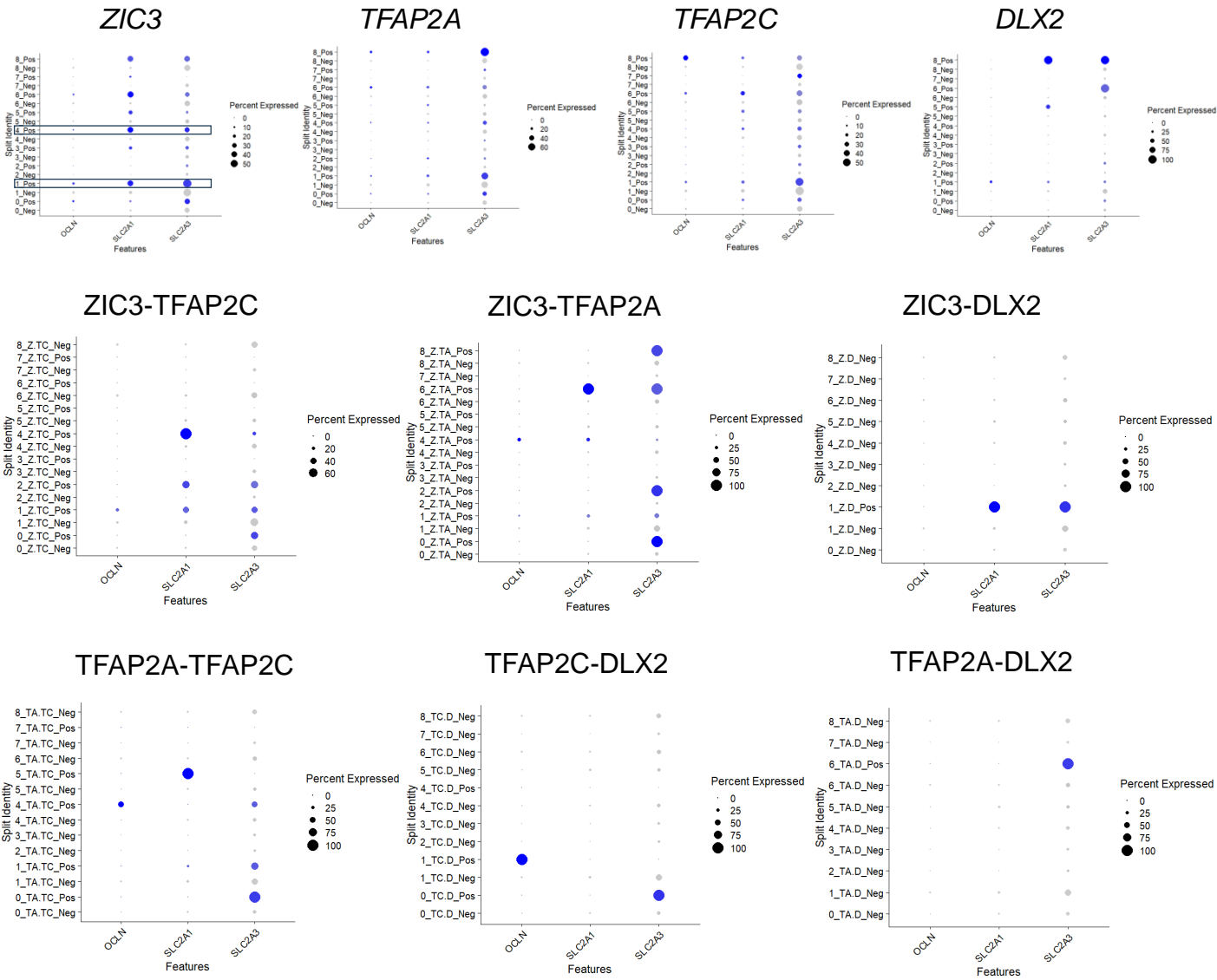

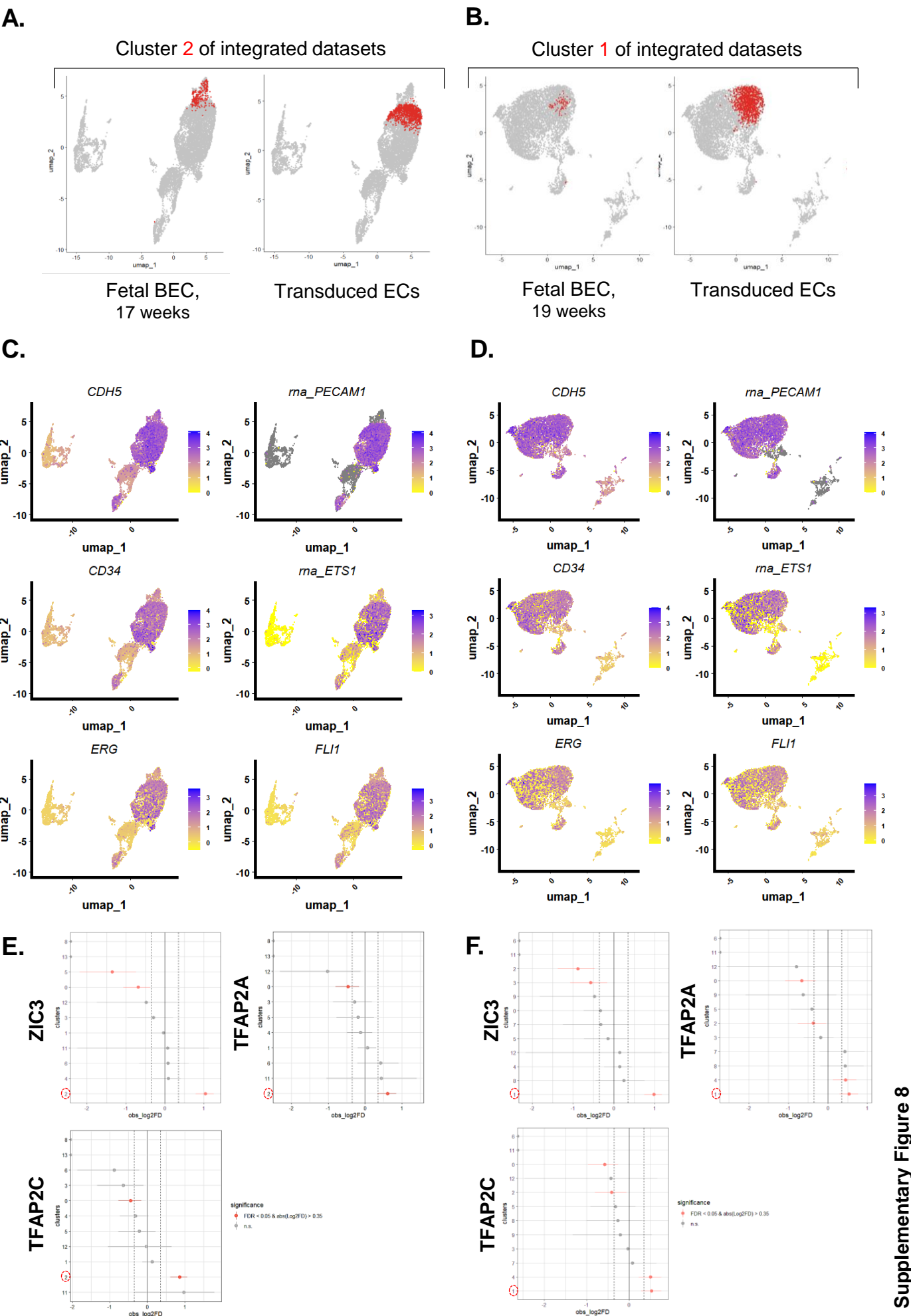

**A.** Cluster 1 of integrated datasets

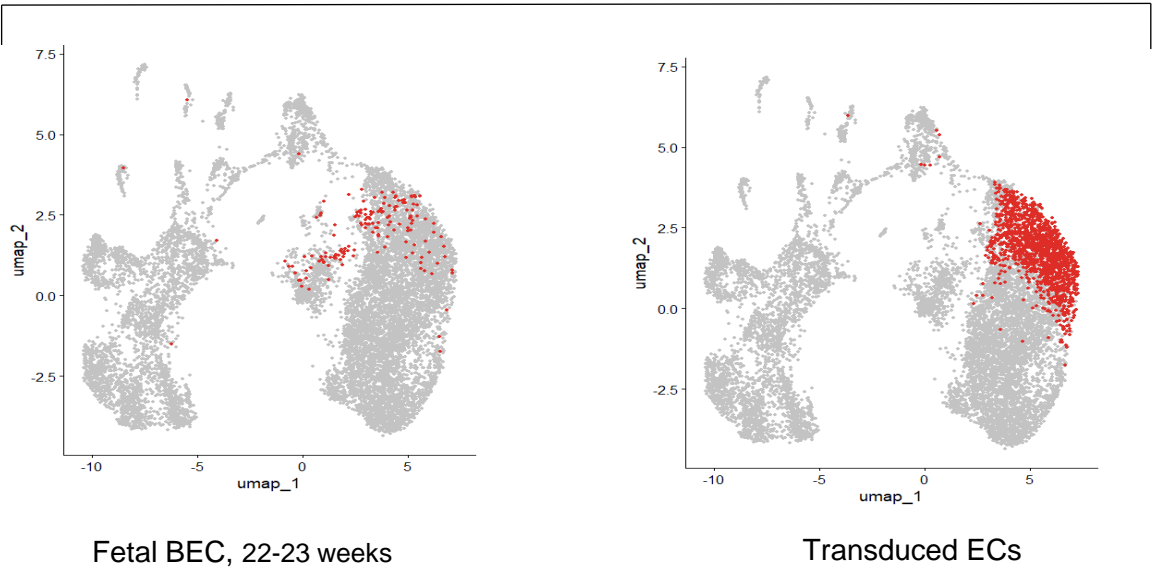

**B.**

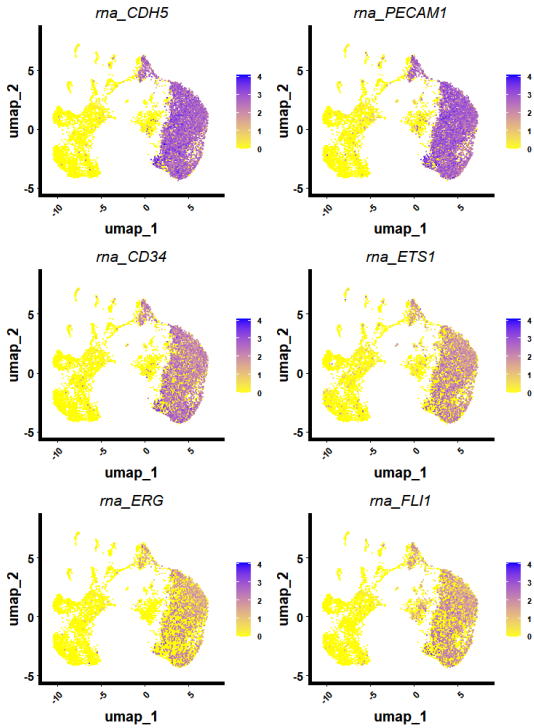

**C.**

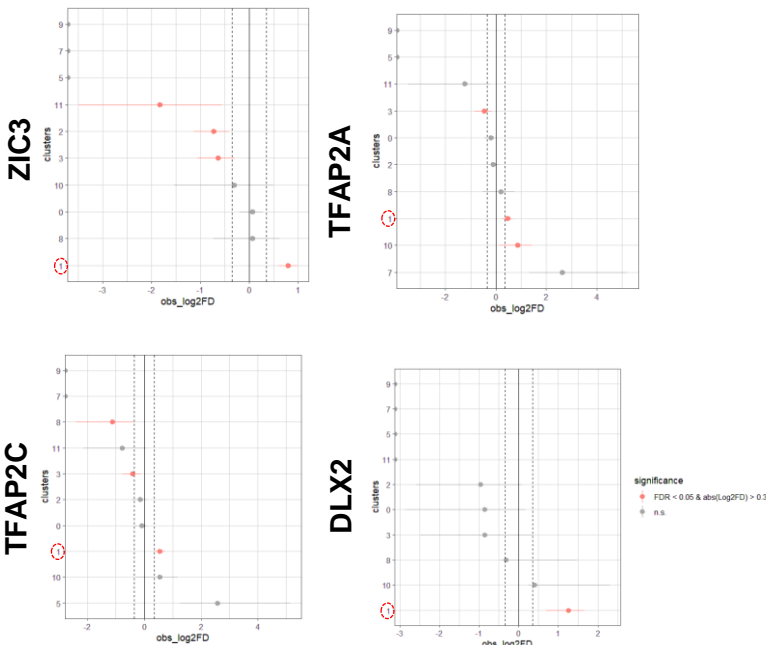

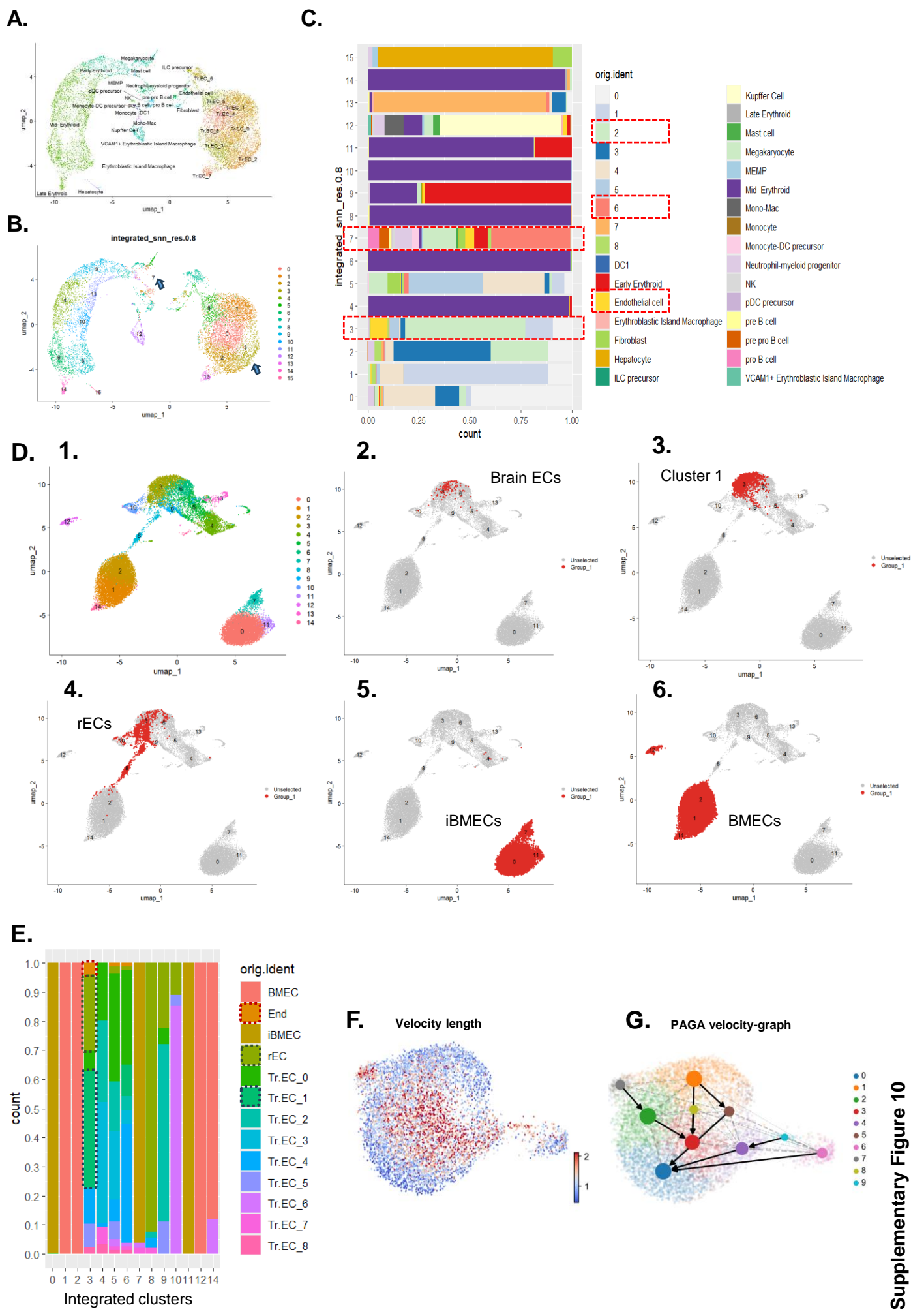

Supplementary Figure 10

Supplement: Supplementary file 1 — Supplementary file1 (PDF 3 MB) [file 12015_2025_10842_MOESM1_ESM.pdf]
