# Supplementary material for: Identification of Cell Fate Determining Transcription Factors for Generating Brain Endothelial Cells
Source: Stem Cell Rev Rep. 2025 Jan 24;21(3):744–66. doi: 10.1007/s12015-025-10842-7 (PMC11965213; doi:10.1007/s12015-025-10842-7)
Supplement: Supplementary file 2 — Supplementary file2 (DOCX 51 KB) [file 12015_2025_10842_MOESM2_ESM.docx]

**Identification of cell fate determining transcription factors for generating brain endothelial cells**

**Stem Cell Reviews and Reports**

**Roya Ramezankhani^1,2,*^, Jonathan De Smedt^1^, Burak Toprakhisar^1,3^, Bernard K. van der Veer^1,4^, Tine Tricot^1^, Gert Vanmarcke^1^, Bradley Balaton^1,4^, Leo van Grunsven^3^, Massoud Vosough^2,5^, Yoke Chin Chai^1^,** **Catherine Verfaillie^1^**

**^1^** Stem Cell Institute, Department of Development and Regeneration, KU Leuven, O&N IV Herestraat 49, 3000, Leuven, Belgium, [roya.ramezankhani@kuleuven.be](mailto:roya.ramezankhani@kuleuven.be)

^2^ Department of Applied Cell Sciences, Faculty of Basic Sciences and Advanced Medical Technologies, Royan Institute, Academic Center for Education, Culture and Research, Tehran, Iran

^3^ Vrije Universiteit Brussel, Liver Cell Biology Research Group, Laarbeeklaan 103, Brussels 1090, Belgium

^4^ KU Leuven Institute for Single Cell Omics (LISCO), KU Leuven-University of Leuven, B-3000 Leuven, Belgium

^5^ Department of Regenerative Medicine, Cell Science Research Center, Royan Institute for Stem Cell Biology and Technology, Academic Center for Education, Culture and Research (ACECR), Tehran, Iran

* Corresponding author

**Supplementary Figure 1. Medium optimization for supporting the culture of iETV2 ECs.**

**A.** Quantitative analysis of key endothelial markers from immunofluorescence staining revealed no major difference between human pluripotent stem cells (hPSC)-iETV2 ECs cultured in liver differentiation medium (LDM) and neural maintenance medium (NMM) (N=1 replicate). **B.** Average expression of *ETV2* gene in brain endothelial cells (ECs) from 6-10 (1), 17, 19 (2), and 22-23 (3) week-old fetal single cell (sc)RNA-Seq datasets. **C-F.** Feature plots of *ETV2* gene expression in the corresponding human fetal scRNA-Seq datasets showing low expression of *ETV2* in human fetal brain of 6-23 weeks old, including the brain EC cluster.

**Supplementary Figure 2. CenTFinder analysis of murine microarray datasets of ECs, including brain ECs.**

**A.** Number of microarrays data per platform and each different types of EC used in the CenTFinder analysis (n=193). LSEC = liver sinusoidal EC. **B.** Up-regulated putative brain EC markers (left) and TFs (right) in the annotated samples against all other ECs. The mean fold change between brain ECs and other ECs is indicated with black dots. Lines span the interquartile range. **C.** Cluster dendrogram demonstrating active modules in at least one of the EC types. Each module consists of clusters of highly correlated genes. **D.** Heatmap of activity scores based on gene set variation analysis (GSVA) for each module and EC subtypes. The colour code of other modules in respect with **C** is as follow: yellow ="CNS development and membrane transport", brown ="Ribosome assembly", turquoise="Nuclear protein (histone) / modification (phosphorylation)", blue ="Oxidative phosphorylation", red ="Lipid metabolism and membrane transport", black ="Cell cycle", magenta="Metabolism and enzymatic activity", greenyellow = "RNA processing", green= "Male gonad development", purple = "Synaptic signalling and transporter activity", midnightblue = "DNA repair", lightgreen = "Vesicles at the ribosomes", cyan = "Ribosomes", lightcyan = "Cytoskeleton, focal adhesions, and cell-substrate junctions", salmon = "Immune response", tan = "Immune response-leukocyte activation", grey60 = "Junctions"). **E.** Gene Ontology (GO) analysis of pink module. **F.** GSVA enrichment scores of each module in samples annotated as brain ECs and all other EC types. **G.** Enriched TF binding motifs are shown for each modules. Y-axis shows the fraction of genes in each module that has binding motifs for related TFs. x-axis shows network enrichment score (NES) based on RcisTarget analysis. Shown TFs are the ones with the highest NES. Top TFs are displayed in red. The orange arrow indicates the *import* module.

**Supplementary Figure 3. Expression of candidate TFs in fetal brain ECs using scRNA-Seq dataset of developing fetuses.**

**A.** Expression of the identified TFs in the *import* module from cenTFinder analysis in fetal brain ECs. **B.** Expression of the final selected 24 TFs as well as ZIC3 in fetal brain ECs (based on (4)).

**Supplementary Figure 4. Marker identification based on merged sc/snRNA-Seq and microarrays datasets.**

**A.** Number of detected genes in each sc/snRNA-Seq dataset. **B.** Dot plot indicating the pool of identified marker genes from individual sc/snRNA-Seq and microarray datasets based on the expression fold change of marker genes in murine brain ECs compared to other ECs and the expression fold change of marker genes in brain EC clusters in sc/snRNA-Seq studies with the highest detected genes. **C.** Identified brain EC-specific marker genes in Allen and Fan *et al.* datasets are ranked based on **1)** at least four-fold change expression in brain ECs from the Allen (5, 6) and Fan *et al.* (3) datasets compared to other cells in sc/snRNA-Seq studies, and at least **2)** four or **3)** two fold change expression in murine brain ECs compared to other ECs and the 90th percentile expression of less than **2)** 7 and **3)** 7.5 fold change in human non-brain ECs compared to brain ECs. **D. 1)** Top 40 gene markers related to capillary bed of brain ECs identified from Kalucka *et al.* (7) **2)** Dot plot indicating the expression fold change of identified markers in capillary bed compared to larger vessels in brain on x-axis and the expression fold change in murine brain ECs compared to other ECs on y-axis. **3)** Box plot indicating the top gene markers of capillary bed compared to larger vessels in brain (Log FC>0), with two fold change expression in murine brain ECs compared to other ECs, the 90^th^ percentile expression of less than 7.5 fold change in human non-brain ECs compared to brain ECs, and at least be expressed in Allen and Fan *et al.* datasets.

**Supplementary Figure 5. Overexpression of 12 candidate TFs.**

**A.** Schematic representation of employed constructs for overexpressing the TFs. **B.** Upon transduction of HEK293 cells with the individual TF encoding vectors, the cells were stained with HA antibody which confirmed the translation of all TFs coding sequence. **C.** Graphic summary of scRNA-Seq strategy upon the transduction of cells with the combination of 12 TFs. (created by Biorender.com)

**Supplementary Figure 6. Cell cluster identification and TF assessment.**

**A.** Gene Ontology (GO) enrichment analysis for biological process (BP) terms and Panther pathway enrichment analysis based on top DEGs of each cluster (P-value <0.05, average log fold change > 0.25) for each cluster. **B.** Expression of a unique gene signature associated with Wnt pathway signaling in cluster 1^T^.

**Supplementary Figure 7.**  **The impact of transduction with the final candidate TFs on the expression of brain EC markers.**

Dot plots indicating expression of brain EC marker genes in transduced cells with at least one of the four final candidate TFs or any double combination of them. Positive (Pos) and negative (Neg) indicate the presence or absence of relevant barcodes for each plot in cluster cells.

**Supplementary Figure 8. Integration analysis with fetal brain scRNA-Seq datasets.**

**A, B.** Uniform Manifold Approximation and Projection (UMAP) of integrated cells with dataset from 17 and 19 week old fetal brain (2). Highlighted cells indicating the cells in cluster 2 and cluster 1 of integrated data which constitutes of fetal brain ECs and a portion of transduced ECs (mostly from cells in cluster 1^T^ of the transduced EC data). **C, D.** Expression of EC markers in integrated data with 17 and 19 weeks old fetuses. **E, F.** Significant enrichment of final TF barcodes in each cluster in integrated data with 17 and 19 week old fetal brains.

**Supplementary Figure 9. Integration analysis with 23-23-week-old fetal brain scRNA-Seq datasets.**

**A.** UMAP of integrated cells with ~23-week-old fetal brain (3, 8). Highlighted cells indicating the cells in cluster 1 of integrated data which constitutes of fetal brain ECs and a portion of transduced ECs (mostly from cells in cluster 1^T^ of the transduced EC data). **B.** Expression of EC markers in integrated data with ~23-week-old fetus. **C.** Significant enrichment of final TF barcodes per each cluster in integrated data with ~23-week-old fetus datasets.

**Supplementary Figure 10. Data integration and RNA velocity analysis.**

**A, B.** UMAP of integrated cells with 9-week-old fetal liver scRNA-Seq dataset (9). The blue arrows in **B** indicating the position of integrated clusters (*i.e.* clusters 3 and 7) which contain most of liver ECs. **C.** Stacked bar plot indicating clusters 3 and 7 of integrated data (on y-axis) which include most of fetal liver ECs and the majority of transduced ECs from clusters 2^T^ and 6^T^). **D.** UMAP of integrated data with BMECs, iBMECs, rECs, (10, 11) and 19-week-old fetal brain data set (2). **D 1-6** demonstrate the location of fetal brain ECs, candidate cluster 1^T^, rECs, cultured BMECs, and iBMECs in integrated dataset. **E.** Stacked bar plot indicating cluster 3 of integrated data (on x-axis) contain the majority of *in vivo* brain ECs as well as cluster 1^T^ and a small portion of rECs. **F.** Velocity length plotted on UMAP indicating the speed of differentiation in each cell cluster. **G.** The PAGA velocity graph. BMEC: Brain microvascular endothelial cell, iBMEC: induced BMEC, rEC: reprogrammed iBMECs with *ETV2*, *ERG*, and *FLI1*

**References**

1. Eze UC, Bhaduri A, Haeussler M, Nowakowski TJ, Kriegstein AR. Single-cell atlas of early human brain development highlights heterogeneity of human neuroepithelial cells and early radial glia. Nature Neuroscience. 2021;24(4):584-94.

2. Jessa S, Blanchet-Cohen A, Krug B, Vladoiu M, Coutelier M, Faury D, et al. Stalled developmental programs at the root of pediatric brain tumors. Nature Genetics. 2019;51(12):1702-13.

3. Fan X, Dong J, Zhong S, Wei Y, Wu Q, Yan L, et al. Spatial transcriptomic survey of human embryonic cerebral cortex by single-cell RNA-seq analysis. Cell Research. 2018;28(7):730-45.

4. Wälchli T, Ghobrial M, Schwab M, Takada S, Zhong H, Suntharalingham S, et al. Single-cell atlas of the human brain vasculature across development, adulthood and disease. Nature. 2024;632(8025):603-13.

5. Bakken TE, Jorstad NL, Hu Q, Lake BB, Tian W, Kalmbach BE, et al. Comparative cellular analysis of motor cortex in human, marmoset and mouse. Nature. 2021;598(7879):111-9.

6. Yao Z, van Velthoven CTJ, Nguyen TN, Goldy J, Sedeno-Cortes AE, Baftizadeh F, et al. A taxonomy of transcriptomic cell types across the isocortex and hippocampal formation. Cell. 2021;184(12):3222-41.e26.

7. Kalucka J, de Rooij L, Goveia J, Rohlenova K, Dumas SJ, Meta E, et al. Single-Cell Transcriptome Atlas of Murine Endothelial Cells. Cell. 2020;180(4):764-79.e20.

8. Garcia FJ, Sun N, Lee H, Godlewski B, Mathys H, Galani K, et al. Single-cell dissection of the human brain vasculature. Nature. 2022;603(7903):893-9.

9. Popescu D-M, Botting RA, Stephenson E, Green K, Webb S, Jardine L, et al. Decoding human fetal liver haematopoiesis. Nature. 2019;574(7778):365-71.

10. Lu TM, Houghton S, Magdeldin T, Durán JGB, Minotti AP, Snead A, et al. Pluripotent stem cell-derived epithelium misidentified as brain microvascular endothelium requires ETS factors to acquire vascular fate. Proceedings of the National Academy of Sciences of the United States of America. 2021;118(8).

11. Lippmann ES, Al-Ahmad A, Azarin SM, Palecek SP, Shusta EV. A retinoic acid-enhanced, multicellular human blood-brain barrier model derived from stem cell sources. Scientific reports. 2014;4(1):4160.
